# Using MEG to Understand the Progression of Light Sleep and the Emergence and Functional Roles of Spindles and K-Complexes

**DOI:** 10.3389/fnhum.2017.00313

**Published:** 2017-06-16

**Authors:** Andreas A. Ioannides, Lichan Liu, Vahe Poghosyan, George K. Kostopoulos

**Affiliations:** ^1^Laboratory for Human Brain Dynamics, AAI Scientific Cultural Services Ltd.Nicosia, Cyprus; ^2^MEG Unit, Department of Neurophysiology, King Fahad Medical CityRiyadh, Saudi Arabia; ^3^Neurophysiology Unit, Department of Physiology, Medical School, University of PatrasRion, Greece

**Keywords:** sleep, spindles, K-complexes, magnetoencephalography (MEG), magnetic field tomography (MFT)

## Abstract

We used tomographic analysis of MEG signals to characterize regional spectral changes in the brain at sleep onset and during light sleep. We identified two key processes that may causally link to loss of consciousness during the quiet or “core” periods of NREM1. First, active inhibition in the frontal lobe leads to delta and theta spectral power increases. Second, activation suppression leads to sharp drop of spectral power in alpha and higher frequencies in posterior parietal cortex. During NREM2 core periods, the changes identified in NREM1 become more widespread, but focal increases also emerge in alpha and low sigma band power in frontal midline cortical structures, suggesting reemergence of some monitoring of internal and external environment. Just before spindles and K-complexes (KCs), the hallmarks of NREM2, we identified focal spectral power changes in pre-frontal cortex, mid cingulate, and areas involved in environmental and internal monitoring, i.e., the rostral and sub-genual anterior cingulate. During both spindles and KCs, alpha and low sigma bands increases. Spindles emerge after further active inhibition (increase in delta power) of the frontal areas responsible for environmental monitoring, while in posterior parietal cortex, power increases in low and high sigma bands. KCs are correlated with increase in alpha power in the monitoring areas. These specific regional changes suggest strong and varied vigilance changes for KCs, but vigilance suppression and sharpening of cognitive processing for spindles. This is consistent with processes designed to ensure accurate and uncorrupted memory consolidation. The changes during KCs suggest a sentinel role: evaluation of the salience of provoking events to decide whether to increase processing and possibly wake up, or to actively inhibit further processing of intruding influences. The regional spectral patterns of NREM1, NREM2, and their dynamic changes just before spindles and KCs reveal an edge effect facilitating the emergence of spindles and KCs and defining the precise loci where they might emerge. In the time domain, the spindles are seen in widespread areas of the cortex just as reported from analysis of intracranial data, consistent with the emerging consensus of a differential topography that depends on the kind of memory stored.

## Introduction

Normal sleep proceeds in 90-min cycles of rapid eye movement (REM) and non-REM (NREM) phases. NREM phase separates further into light (NREM1 and NREM2 stages) and slow wave (NREM3 and NREM4 stages) sleep (Rechtschaffen and Kales, 1968). NREM2 occupies about half of our sleeping time. Although, loss of consciousness can be variously documented at NREM1 (Ogilvie, 2001) the definitive start of sleep is practically marked by the first appearance of the hallmarks of NREM2 (Cvetkovic and Cosic, 2011), spindles and K-complexes (KCs). A spindle is a brief 11–16 Hz oscillation (De Gennaro and Ferrara, 2003), while a KC consists mainly of a prominent negative wave, the largest waveform of healthy EEG (Cash et al., 2009). Several recent studies have used source analysis to estimate neural generators of EEG and MEG signals during spindles and KCs. Most commonly, spindle-related activity is found in medial parietal, central and frontal areas (Manshanden et al., 2002; Ishii et al., 2003; Urakami, 2008; Gumenyuk et al., 2009; Dehghani et al., 2010a); KC-related brain activity has been identified in frontal cortical areas, along the cingulate gyrus, the precuneus, and the insula (Murphy et al., 2009), as well as in deep central temporal (Yoshida et al., 1996) and parietal areas (Lu et al., 1992; Numminen et al., 1996). These and other studies using intracranial recordings (Wennberg, 2010; Andrillon et al., 2011; Peter-Derex et al., 2012; Frauscher et al., 2015) and hemodynamic methods (Larson-Prior et al., 2009; Maquet, 2010; Caporro et al., 2012) have identified a wide range of brain areas showing high activation in the time periods of spindles and KCs, but no clear-cut hints about the underlying mechanisms that are responsible for their generation.

At the neurophysiological level, we know a lot about how neurons behave during spindles (De Gennaro and Ferrara, 2003; Llinás and Steriade, 2006) and KCs (Colrain, 2005; Cash et al., 2009) and evidence accumulates about their multiple and important roles in sleep and sleep-mediated brain functions (Khazipov et al., 2004; Colrain, 2005; Cash et al., 2009; Diekelmann and Born, 2010; Andrade et al., 2011; Halasz and Bodizs, 2013; Stickgold and Walker, 2013). In contrast, we know little about the evolution and continuity of large scale brain activity patterns leading to the safe emergence of spindles and KCs (Alloway et al., 1999; Gross and Gotman, 1999; De Gennaro and Ferrara, 2003; Colrain, 2005; Larson-Prior et al., 2009; Maquet, 2010; Andrillon et al., 2011; Dehghani et al., 2011b; Kokkinos and Kostopoulos, 2011; Caporro et al., 2012; Kokkinos et al., 2013). Early PET and fMRI studies found localized decreases during NREM sleep compared to wakefulness (Czisch et al., 2004; Kaufmann et al., 2006; Wehrle et al., 2007). In recent years EEG triggered fMRI is increasingly used making possible to focus the analysis of both EEG and fMRI on periods with specific EEG characteristics of NREM. In one such study regionally specific increases in activity were identified for spindles in thalami, posterior cingulate, insula and sensorimotor cortices. K-complexes corresponded to increased signal in thalami, superior temporal lobes, paracentral gyri, medial regions of the occipital, parietal and frontal lobes. Regions of decreased signal were not found (Caporro et al., 2012).

The imaging studies converge with the electrophysiological ones to the view of NREM sleep not as a state of brain quiescence, but as a highly active state during which brain activity is consistently synchronized to slow waves and spindles in specific brain regions. These segregated activations are in line with the local nature of spindles and slow waves demonstrated by recent electrophysiological data (Nir et al., 2011). Furthermore, they are consistent with the notion that spindles emerge when transmission of sensory information to the cortex is minimal, blocking perception and thus isolating the brain from external disturbances, while slow waves like K-complexes reflect an enhanced processing of external information (Dang-Vu, 2012).

Sleep starts due to confluence of the phase of circadian pacing and the metabolically driven homeostatic factors accumulating during wake state. These two factors acting through hypothalamic GABAergic neurons in ventrolateral and medial preoptic nuclei inhibit the arousing centers that are located mainly in the brainstem; the inactivation of these arousing centers leads to sleep onset. The mutual inhibition between the hypothalamic sleep promoting and the brainstem arousing system sets up the conditions for a flip-flop switch that ensures rapid and complete transitions between wake and sleep, while an analogous mutual inhibition process between REM-on and REM-off brainstem and diencephalic neurons regulates the sleep macrostructure in about 90 min cycles of NREM and REM phases (Saper and Sehgal, 2013).

During each sleep stage, periods with large graphoelements (i.e., elementary waveforms visually identified in raw EEG/MEG as salient single or periodic events) and oscillations in the electroencephalogram (EEG) are interspersed with relatively quiet, “core” periods defined as “low amplitude segments of data with no large EEG events and clearly separated from high voltage graphoelements of each sleep stage (e.g., NREM2 spindles and KCs)” (Ioannides et al., 2009). These core periods correspond to the shorter lasting phase “B” of the cyclic alternating pattern in NREM (Terzano et al., 2001; Ferri et al., 2006; Wehrle et al., 2007). The sleep stages of light sleep (NREM1 and NREM2) and slow wave sleep (NREM3 and NREM4) are defined by the imprints on the EEG of highly rhythmic and/or large amplitude graphoelements that define each sleep stage. However, the sequence of graphoelements does not reveal any principled change, neither within nor across sleep stages. Drawing partly from our previous results (Ioannides et al., 2009), we propose a twin hypothesis as an aid for searching for a principled evolution in the EEG, within and across sleep stages. First we propose that the highly rhythmic and high amplitude events that define each sleep stage are only the end products of more fundamental changes that are not so obvious in the time domain traces of the EEG, *especially during* the periods of these high amplitude events. Second, we propose that if there exist fundamental mechanisms that generate the regular sequence of succession of NREM1 to NREM2 and then to slow wave sleep then the signatures of these mechanisms will be still in evidence well away from the large graphoelements as changes in the spectral properties of the quiet periods of each sleep stage. We test this hypothesis by setting up the analysis to hunt for two types of regional spectral changes: the first type is circumscribed regional spectral changes marking the activation/deactivation of control and/or initiator areas that drive these changes. The second type are widespread spectral changes, again at specific frequencies, that are the outcome of the spreading influences from the initiator areas resulting in shifts in overall arousal and mode of operation of large brain areas. Assuming we found evidence for these regional spectral signatures, we further hypothesized that the emergence of spindles and KCs that so far appears random may emerge as their continuation: the changes in core states (from the period before sleep to NREM1 and then NREM2) will intensify on the approach and culminating into the apparent wild excursions during spindles and KCs. We further anticipate that a differentiation will be found in the sequence of core period spectra during the few seconds before spindles and KCs. This differentiation in approach period could provide valuable clues about the respective roles of spindles and KCs by revealing specific brain areas, with known specialization, that must be activated or stopped according to the specific task(s) that evolution has designed spindles and KCs to do.

## Materials and methods

Our analysis makes full use of the rich information in the raw MEG signal applying only the minimal necessary pre-processing (Section Data Acquisition and Pre-Processing). Each time slice of each single trial of the raw (minimally processed) MEG signal is then submitted to an independent magnetic field tomography (MFT) analysis (Ioannides et al., 1990; Taylor et al., 1999). The output is millisecond-by-millisecond tomographic description of the primary current density throughout the brain. The basic algorithm and the mathematical foundation of MFT have been described in detail in dedicated publications (Ioannides et al., 1990; Taylor et al., 1999) and the key points summarized in many of the major studies, e.g., (Poghosyan and Ioannides, 2008) and reviews (Ioannides, 2006), including our previous two sleep studies (Ioannides et al., 2004a, 2009). The power of MFT analysis draws from the fact that the full information in the data is utilized: the analysis is applied independently time-slice by time-slice on minimally processed raw MEG data; the specific questions of each new study are addressed by adding new refinements in the post-MFT statistical analysis of the single trial tomographic solutions.

**Figure 1:**
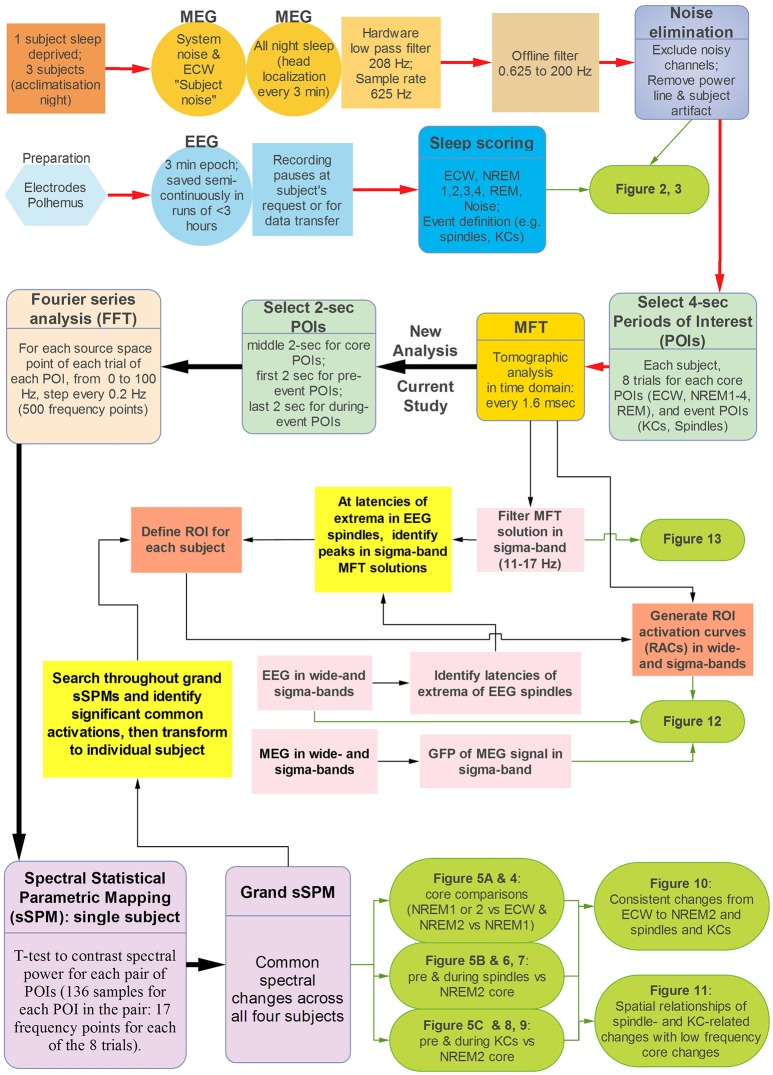
The analysis steps used in this paper. The red path, traces the first steps of the analysis as described in Sections Subjects and Overall Experiment Planning, Data Acquisition and Pre-Processing, Displays of the Raw MEG Signal and Auxiliary Channels, and Source Reconstruction. For this early part of the analysis the same methods are used as in our previous two sleep studies, so only the key steps are included more details can be found in Figure 1 of Ioannides et al. (2009). The next two figures with results of this paper (Figures 2, 3) summarize aspects of the recordings (Figure 2) and properties of the EEG and MEG signal (Figure 3). The spectral analysis of the MFT solutions (described in more detail in Sections Fourier Analysis of MFT Solutions, Spectral Statistical Parametric Mapping (sSPM) for Each Subject, and Grand sSPM and Reporting of Statistical Significance) constitutes the main part of the new analysis and it is marked by the black paths with heavy arrows, continuing (from right to left) after the MFT box, with final output Figures 4–11 and Table 1. The parts linked with light black arrows show analysis steps that combine old and new methods (see also Section Regions of Interest and Time Domain Analysis) to produce the last two figures of this paper (Figures 12, 13). These two figures provide a bridge between the results from two distinct types of analysis. In the first, the emphasis is placed on (the peaks of activity in) the time-domain while in the second the emphasis is placed on (spectral properties of regional activity in) the frequency domain. To achieve this integrative role the analysis (and results in Figures 12, 13) combine information from the raw EEG and MEG signals, the MFT tomographic solutions and the grand sSPM results showing the patterns of both raw signal and MFT solutions across widely different timescales from milliseconds to minutes.

In the subsections below we will highlight the methods used in this, our third sleep study, with references to the detailed description in our two earlier sleep studies and other recent MFT publications. We will clearly specify the departures from the methods previously employed and the motivation for each one. The overall sequence of operations involved in the methods is summarized in Figure 1 that is used as a cross-reference back to the methods in our two previous studies, and forward to the results and figures reported in the next sections.

### Subjects and overall experiment planning

Four healthy right-handed male subjects (with ages at the time of the experiment 25, 30, 31, and 49) participated in the sleep experiment, after a night of acclimatization. For the details of the sleep protocol and the acclimatization night see (Ioannides et al., 2004a) and accompanying supplementary material. RIKEN's (the institution where the experiment was conducted) ethics committee approved the study, and all the subjects gave their informed written consent after all procedures were explained to them before the experiment.

**Table 1:** Key focal activations.

**ROI #**	**Name**	**BA**	**Figure or NI2009 where ROI appears**	**Talairach coordinates (mm)**		
				**x**	**y**	**z**
1	Frontal Pole	10/11	4, 5A, 10, 11	0	49	−14
2	rACC	24/32	4, 5A, 5B	0	33	15
3	Anterior MPFC	9/10	4, 5A	0	53	31
4	DMPFC	6/8	4, 5B, 10, 11	0	17	45
5a	DMPFC	8/9	NI2009	0	54	28
5b	L-DMPFC	8/9	NI2009	−5	42	31
5c	L-Precuneus	7	NI2009	−5	−62	51
5d	L-DLPFC	9	NI2009	−36	27	36
5e	Left SPL	7/5	NI2009	−12	−55	57
6	Precuneus	7	4, 5A, 5B, 11	0	−61	38
7	MCC	24	4, 5B, 5C, 11	0	0	34
8	sgACC	25	4, 5B, 6, 11	0	6	0
9	LC		4, 5A	0	−26	−13
10	Left DLPFC	8/9	6, 7, 10, 11	−24	27	30
11	Left OFC	11	7, 9, 10,11	−11	22	−14
12	Left NBM	47	11,12	−25	6	−9
13	Left Precuneus	7	7, 11	−8	−61	46
14	Left SPL	7	11, 12	−12	−68	41
15	Left MCC	32/6	12	−11	14	40
16	Left preCG	6	12	−53	−1	36
17	Right MCC	32/6	12	12	12	41
18	Right SPL	7/19	12	15	−74	36
19	Right NBM		12	14	7	−10

*Centroids of key focal activations identified from sSPMs and during spindles, together with their Talairach coordinates and the figures where they appear (usually as focal changes in spectral power). The coordinates for the set 5a–e (NI2009) are taken ref. (Ioannides et al., 2009), and they correspond to areas of increased gamma band activity during NREM2 core (5a) or REM core (5b–e) compared to the eyes closed condition before sleep. The other areas are from the current study: rACC, rostral anterior cingulate cortex; MPFC, medial prefrontal cortex; DMPFC, dorsal MPFC; MCC, midcingulate cortex; sgACC, subgenual ACC; LC, locus coeruleus; DLPFC, dorsal lateral prefrontal cortex; OFC, orbitofrontal cortex; NBM, nucleus basalis of Meynert; SPL, superior parietal lobule; preCG, precentral gyrus. The dorso-caudal anterior cingulate of reference (Voysey et al., 2015) corresponds to MCC, left MCC and right MCC*.

To perform a whole night MEG sleep study, we introduced new features to the MEG acquisition system and added special procedures in the acquisition and data storage hardware and software, for details see (Ioannides et al., 2004a) and its supplementary material. We also made every effort to make the sleep as comfortable and natural as possible in the demanding conditions of performing an MEG recording allowed. Minimizing subject discomfort was aided by allowing a fully supine sleep throughout the night, allowing some movement and self-adjustment of the head in the helmet and making possible for subjects to interrupt the experiment any time they felt uncomfortable (usually because they needed to use the toilet). To make sure that all the segments of data analyzed were well away from head movements (voluntary or involuntary), we partitioned the entire nights recordings into 3 min segment and selected segments for analysis that were free of large artifacts and movements exceeding 3 mm (typically the head movement in the selected segments was below 2 mm). More precisely, the continuous recording was interrupted at two planned stages and further stoppages were allowed on demand by the subject. The first planned interruption was every 3 min, to record the subject's head location relative to MEG sensors, using head localization coils attached to the subject's head. The previous recording of the head localizations and the one at the end of the 3 min were compared; if they were within the set criterion (3–4 mm), the segment was inspected for large noise components and if none was found the segment was selected as candidate for further analysis. For each segment selected for further analysis a separate co-registration of the MEG sensors with the individual high-resolution anatomical MRIs was performed using a standard procedure described in Ioannides et al. (2004a). The second planned interruption was every 3 h or so, to download the recorded data from the temporary disk (with limited capacity) attached to the acquisition system to a more permanent storage. The recording could also be interrupted by a request from the subject, and three of our subjects used this option to use the toilet. The last interruption could be a long one because the EEG electrodes had to be removed and re-applied and the entire initial testing of the acquisition repeated.

### Data acquisition and pre-processing

MEG was recorded throughout the night using a 151 gradiometer whole-head system (CTF/VSM Omega System, Canada) at a sampling rate of 625 Hz and low pass filter at 208 Hz. The following auxiliary channels were recorded in synchrony with the MEG: scalp EEG from C3 and C4 locations referenced to A2 and A1, respectively, vertical and horizontal EOG and electromyogram (EMG) from the chin. The EEG and EMG channels were pre-processed independently of the MEG using filters appropriate for sleep scoring and/or event identification. We stress that the purpose of the experiment was to collect an excellent MEG record of whole night sleep; we used the minimum number of EEG channels needed for professional sleep scoring and avoided to add more electrodes to limit noise interference in the MEG and discomfort for the subject.

As can be seen in Figure 1, the pre-processing leading to the time-domain MFT analysis was identical to that of our second sleep study (Ioannides et al., 2009). The minimal pre-processing involved removal of noisy channels (typically 0, 1, or 2 MEG channels), Independent Component Analysis (ICA) to remove eye and cardiac artifacts, and large external noise or body movement artifacts (with no obvious head movement). In addition to the pre-processing in the first sleep study, offline, the MEG signal was converted to a 3rd order synthetic gradient and band-pass filtered from 0.625 to 200 Hz, with notch filters at 50 Hz and its harmonics. Commonly used procedures were employed to score the sleep stages (Rechtschaffen and Kales, 1968). For details on sleep stage scoring and identification of core periods see (Ioannides et al., 2004a).

For the NREM2 hallmarks we defined the “before” spindle or KC periods of interest (POI) as the first 2 s that contained no obvious KC or spindle or other large events. The “during” spindle or KC POIs were defined as the last 2 s that contained either a spindle or a KC beginning at its onset. We maintained the same length for all POIs, defining the POI core periods as the 2-s periods in the middle of each identified low-amplitude 4-s data segments for each trial of each condition, i.e. for the eyes closed waking (ECW) period before sleep onset, and the NREM1 and NREM2 core periods.

### Displays of the raw MEG signal and auxiliary channels

**Figure 2:**
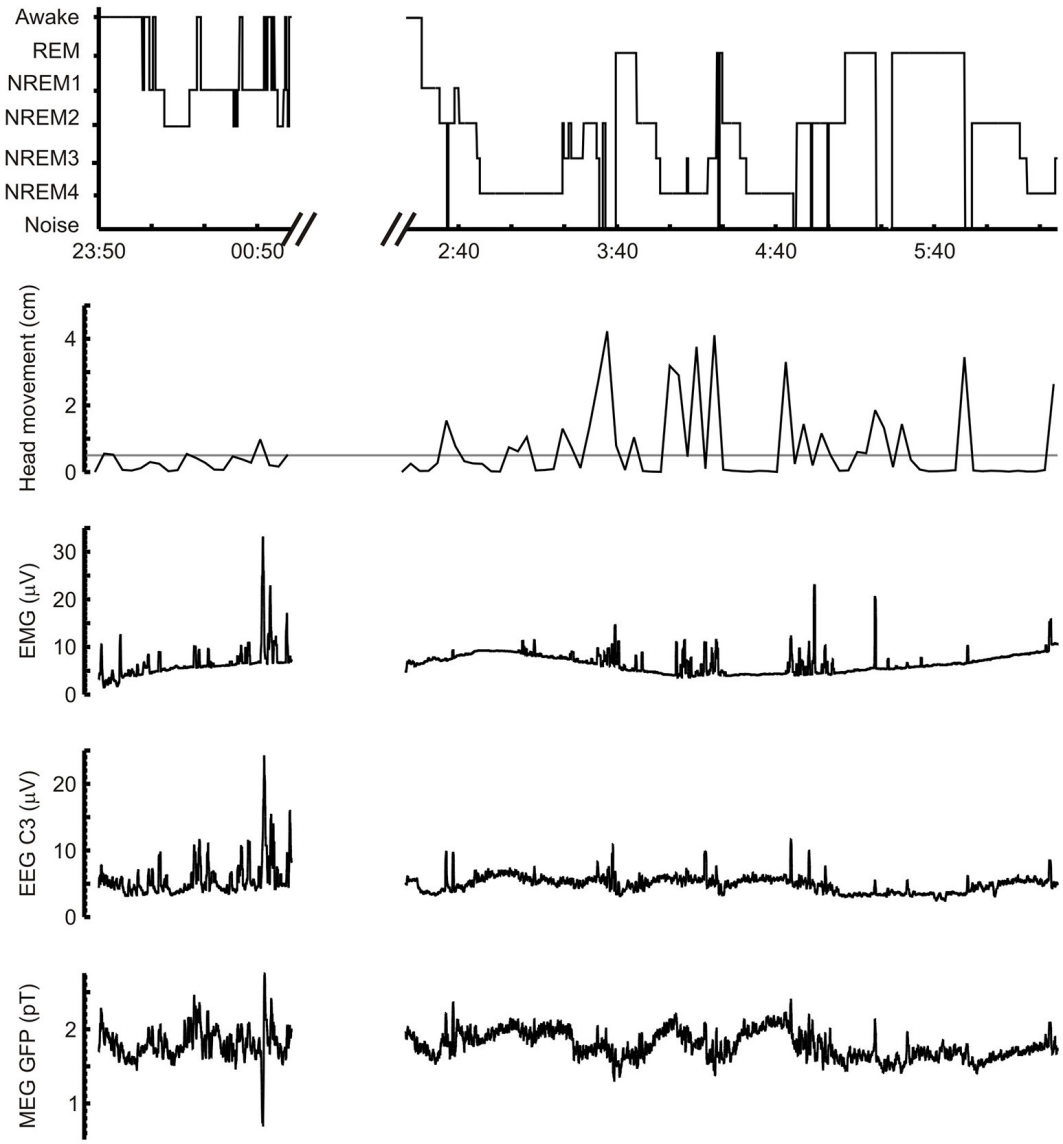
Typical example of full night's recording. Five and half hours of one subject's sleep, from 23:50 in the evening to 01:00 the following morning and from 2:20 to 6:20. The break in the recording between the two segments was made after the subject requested to stop the recording to use the toilet; most of the break time is needed for removing and re-placing electrodes and re-checking all acquisition setup. Note how quickly the subject falls asleep after the recording is resumed. The five graphs from top to bottom show: hypnogram of sleep stages, head movements (in cm) during recording (the gray horizontal line marks the 5 mm threshold of selecting segments for detailed analysis), the EMG signal (submental electrode), the EEG signal from C3 electrode and the MEG global field power (GFP) time course. All three electrophysiological signals were smoothed with a 30 s running window after filtering in the 5–98 Hz (EMG) and 3–45 Hz (EEG and MEG) bands. The EMG and EEG signals were rectified after filtering and before smoothing.

**Figure 3:**
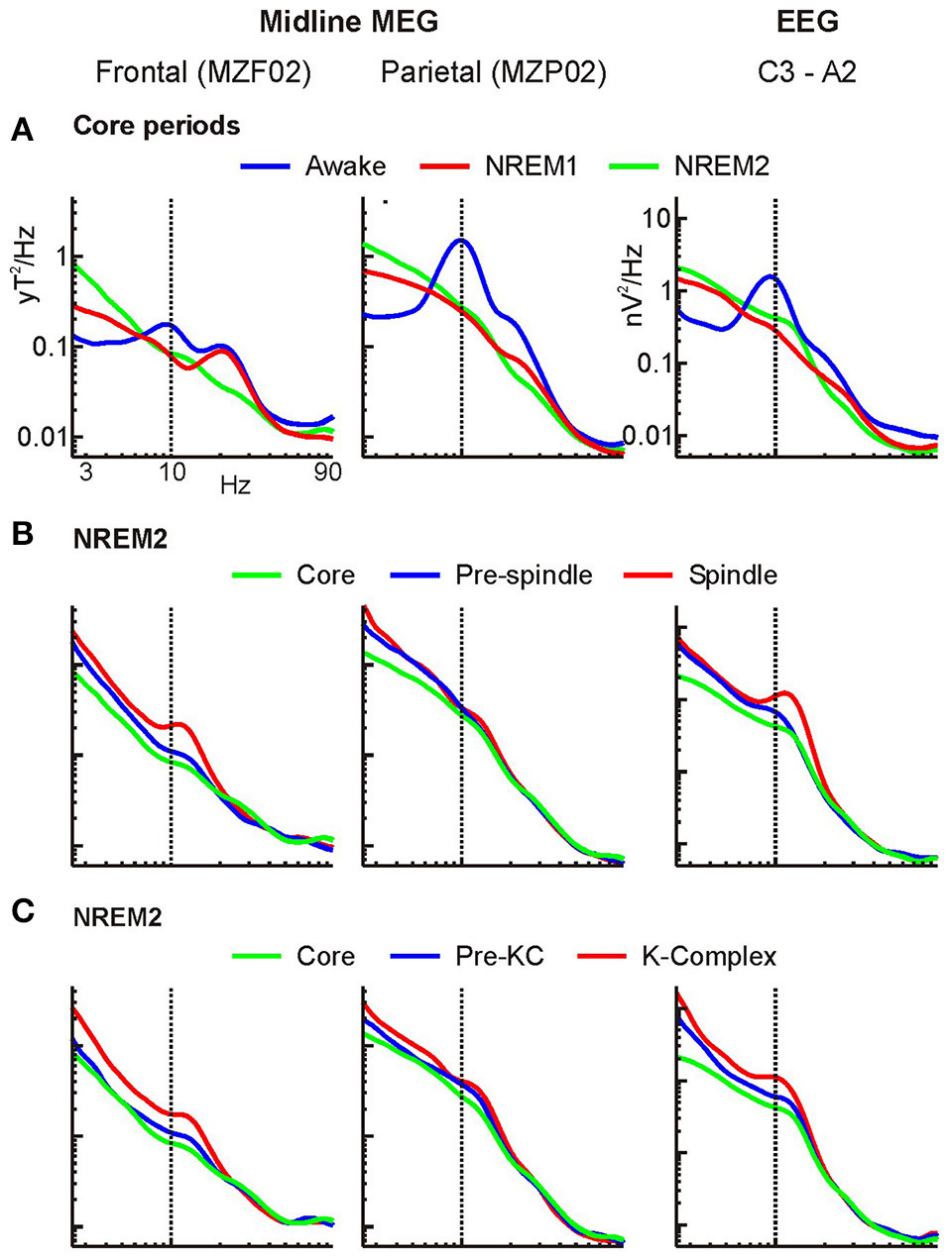
Grand average MEG and EEG power spectra. Spectra for a frontal and parietal midline MEG channels and an EEG channel. Logarithmic scale is used on both the frequency and power axes (log-log plot). **(A)** Comparison of core periods. **(B,C)** Comparison of NREM2 core period with periods immediately before and during **(B)** spindles and **(C)** KCs. The NREM2 core period spectra (green curves) are shown in all figures. For ease of comparisons the same frequency axis (abscissa) is used in all 9 plots, and the same power axis (ordinate) is used in all MEG (first two columns) plots and in all EEG (third column) plots.

A grand summary of the sleep history and awakening events during the night for one subject is furnished in Figure 2 of the results plotting together on the same scale the hypnogram and key measurements derived from the movement, EEG, EMG, and MEG sensors. We used Fourier series analysis to compute the power spectrum of MEG and EEG sensor signals from each trial (56 trials per subject). For each POI, the spectra across trials and subjects were averaged to obtain the grand-average power spectra for each EEG electrode and the global field power (GFP) of the ensemble of MEG channels. The result is summarized in Figure 3 in the Results Section.

### Source reconstruction

Source analysis of MEG signals for each trial was performed using MFT (Ioannides et al., 1990; Taylor et al., 1999). MFT estimates the three-dimensional distribution of current density vectors throughout the brain independently for each time sample (1.6 ms step) of each trial; for each trial the output is therefore a spatiotemporal description that can be viewed as a sequence of spatial maps, or a collection of independent time series computed by sampling across time the continuous MFT estimates at regular grid points. For each time slice of each single trial, the continuous estimate of the current density vector is sampled and stored at regular intervals, spaced about 8 mm across and refer to for simplicity hereafter as voxels. This approach is possible thanks to the power of the non-linear algorithm of the MFT method that is particularly robust to noise and has optimal properties for tomographic analysis (Ioannides et al., 1990; Taylor et al., 1999). For a summary of the basic concepts underlying MFT see (Ioannides et al., 2004a; Poghosyan and Ioannides, 2008).

In the current study we have identified neural activity in a number of deep brain structures. For many years, it was assumed that MEG is insensitive to such deep sources, because of their distance from the sensors (magnetic field decays with the square of distance from a current source) and complex neuronal architecture (the magnetic fields produced by inconsistently oriented neurons cancel rather than summate; Hillebrand and Barnes, 2002; Attal et al., 2009). While this traditional assumption has been supported by some studies (Guy et al., 1993; Mikuni et al., 1997; Shigeto et al., 2002; de Jongh et al., 2005; Ossenblok et al., 2007; Hunold et al., 2016), there is a growing body of recent literature clearly demonstrating that MEG can detect and localize deep brain sources, when appropriate models and methods are used (Ribary et al., 1991; Tesche, 1996; Gross et al., 2001; Hillebrand and Barnes, 2002; Stephen et al., 2005; Papadelis et al., 2009, 2012; Riggs et al., 2009; Attal et al., 2012; Attal and Schwartz, 2013). Moreover, the source localization methodology employed here, has been used to successfully identify deep brain activity in a number of earlier studies (Ribary et al., 1991; Ioannides et al., 2004a,b, 2005).

### Fourier analysis of MFT solutions

In our previous two sleep studies, we used the MFT solutions in the time domain to compare the power at different times (e.g., before and during eye movements and other large graphoelements), confirming the ability of MFT to localize activity in single trials even at deep structures like the pontine nuclei (Ioannides et al., 2004a). In our second sleep study, we used the time domain MFT solutions, but performed the statistical analysis after filtering them in the wide (0.624–200 Hz) and gamma (25–90 Hz) bands. The analysis was particularly successful when the core periods were contrasted with the awake state. In addition to the clear results for the gamma band, the analysis showed large scale changes in low frequency components for the transitions from awake state to the NREM1 and NREM2, as these were reflected in the statistical comparisons of MFT solutions for wide band core state activity with the awake state.

The key difference of the current study compared to the previous two studies is that the Fourier analysis was performed on the MFT solutions, producing spectral (frequency) tomographic representations for each trial. Specifically, at each voxel in the brain, time series for each component of the current density vector (the main output of the MFT analysis) was Fourier transformed to the frequency domain. Each trial was then represented by 500 vector spatio-frequency maps, with each map representing the current density vector of a given frequency in the range 0–100 Hz, with a step of 0.2 Hz. For the only other example of similar Fourier analysis of time domain MFT solutions see (Ioannides et al., 2013).

### Spectral statistical parametric mapping (sSPM) for each subject

The *t*-test was used to contrast the spectral power (modulus of the current density vector) for each pair of POIs at a (center) frequency, f, within a frequency band of width 3.2 Hz for the 8 trials of each POI of the pair. The center frequency started at 1.6 Hz and increased to 97.6 Hz with a step of 1.6 Hz. The distribution for each POI at each center frequency was made up of the 17 distinct spectral values within the 3.2 Hz wide band, e.g., for *f* = 4.8 Hz, the 17 values were 3.2, 3.4, …, 6.2, 6.4 Hz. We used the *t*-test to contrast each core period with the awake resting state (ECW), NREM2 with NREM1 core periods, and the spectral activity before and during the spindles and KCs with the NREM2 core periods. We will report the results of the statistical comparison at five frequency bands: delta (*f* = 4.8 Hz; band from 3.2 to 6.4 Hz), theta (*f* = 6.4 Hz; band from 4.8 to 8 Hz), alpha (*f* = 9.6 Hz; band 8 to 11.2 Hz), low sigma (*f* = 11.2 Hz; band from 9.8 to 12.8 Hz), and high sigma (*f* = 14.4 Hz; from 12.8 to 16 Hz). For the delta band we chose 3.2 rather than 1.6 Hz for the center frequency to avoid the edge of the high pass filter and the low frequencies (below 1 Hz), where the shielded room is less effective in eliminating external noise. We also repeated the entire analysis, using different selections of core periods (the first and last 2 s of the original 4 s segments); the new comparisons produced nearly identical results (not shown) demonstrating that the differences between core states were robust and not the artifact of selection biases.

### Grand sSPM and reporting of statistical significance

**Figure 4:**
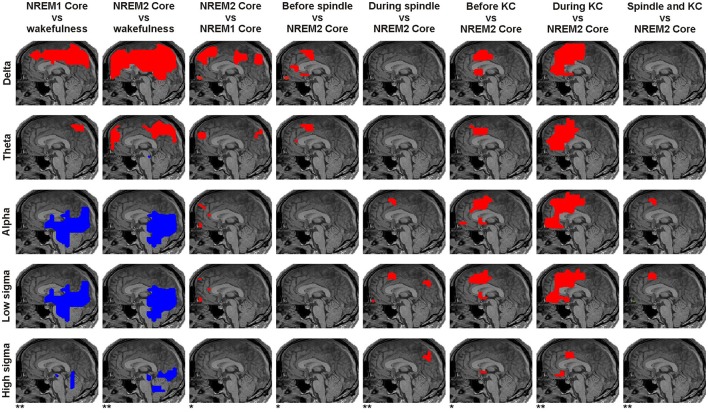
Panoramic overview of the changes in activity along the midline sagittal cut. Each column shows the result for a different comparison, specified on the title at the top of the column. The changes displayed for each comparison represent common (for all subjects) increases (red) or decreases (blue) in spectral power at the significance level indicated at the bottom of the column: modest changes (*p* < 0.05) are denoted by one asterisk (^*^); prominent changes (*p* < 0.00001) are denoted by a double asterisk (^**^). Each row represents the result for a different frequency band as printed (vertically) at the beginning (left) of each row.

**Figure 5:**
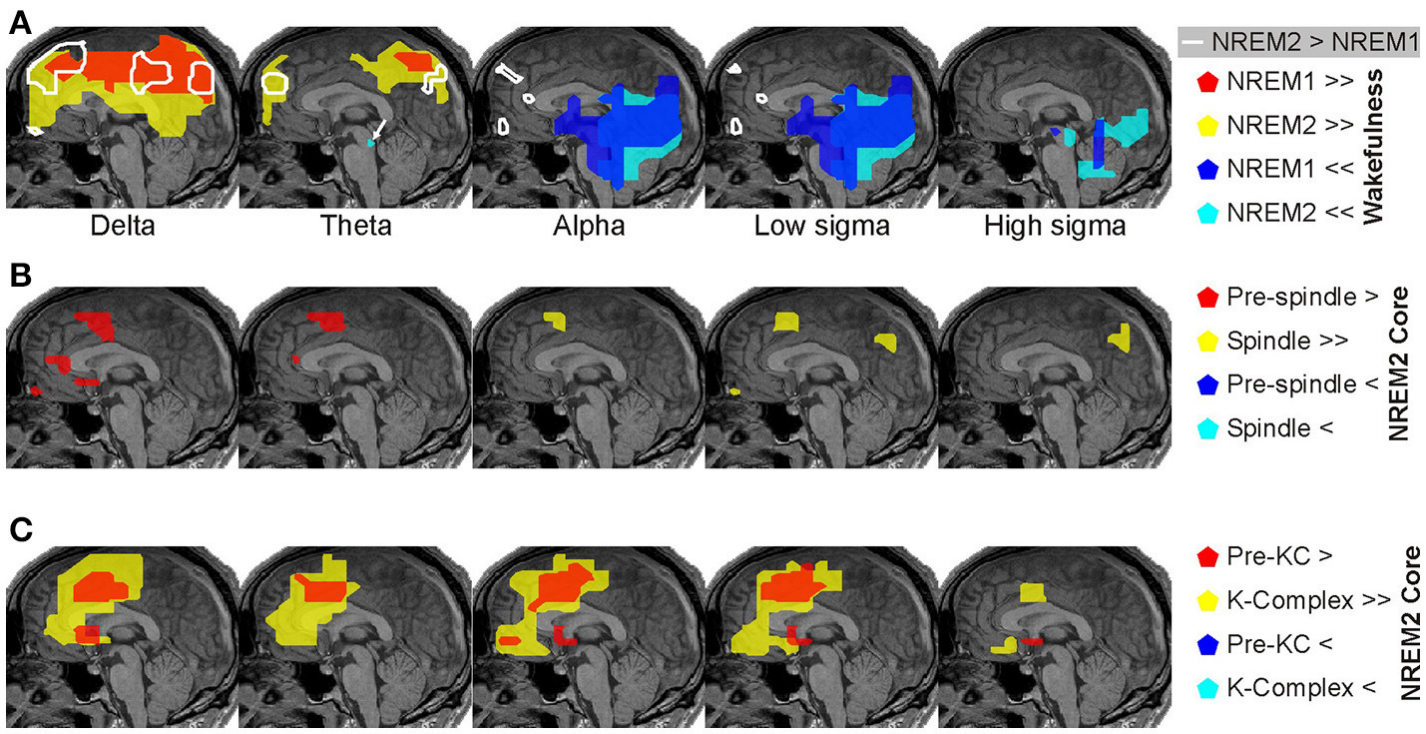
Spectral power changes close to the midline saggittal slice across the periods of interest (POI). Spectral power changes common across all subjects are shown. **(A)** Prominent (*p* < 0.00001), increases (red and yellow) and decreases (blue and cyan) of brain activity in NREM1 (red and blue) and NREM2 (yellow and cyan) core periods from the awake resting state baseline are shown on a midline sagittal MRI slice. White contours encompass regions with modest (*p* < 0.05) increases in activity obtained from direct comparison of NREM2 with NREM1 core periods (NREM2 > NREM1). **(B)** Significant increases of brain activity immediately before (*p* < 0.05; red) and during (*p* < 0.00001; yellow) spindles. At a lower significance threshold of *p* < 0.05, a wider set of areas was identified during spindles in the cortex and upper brainstem and thalamus (Figures 6, 7). **(C)** Significant increases of brain activity immediately before (*p* < 0.05; red) and during (*p* < 0.00001; yellow) KCs. Note that neither for spindles nor for KCs we find any reduction in spectral power relative the NREM2 core period. The statistically significant activations at the thresholds of *p* < 0.00001 and *p* < 0.05 are referred to as “prominent” and “modest” in the manuscript and indicated by the symbols >>/<< and >/< in figures, respectively. For details away from the midline see Figures 6–9.

**Figure 6:**
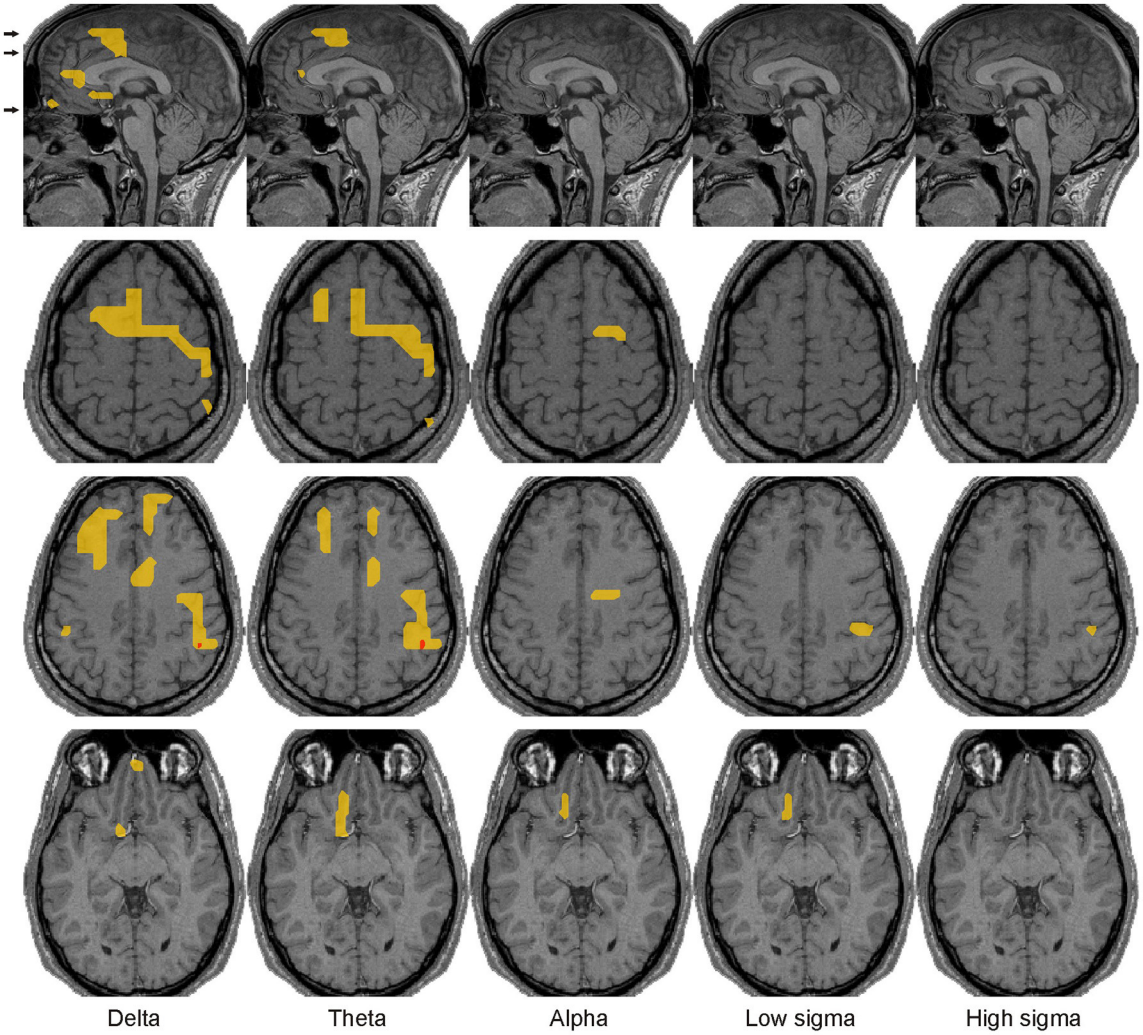
Spectral power changes before spindles. Common across all subjects increases of brain activity before spindles over NREM2 core periods at the significance thresholds of *p* < 0.00001 (red) and *p* < 0.05 (yellow) are shown on the midline sagittal slice and three axial slices. The positions of the axial slices are indicated by black arrows on the sagittal view. The last row shows that the middle part of the elongated left OFC area, lying within or close to the NBM, is the only basal forebrain area that maintains the increase in spectral power in the POI before spindles relative to NREM2 core in the alpha and low sigma bands.

**Figure 7:**
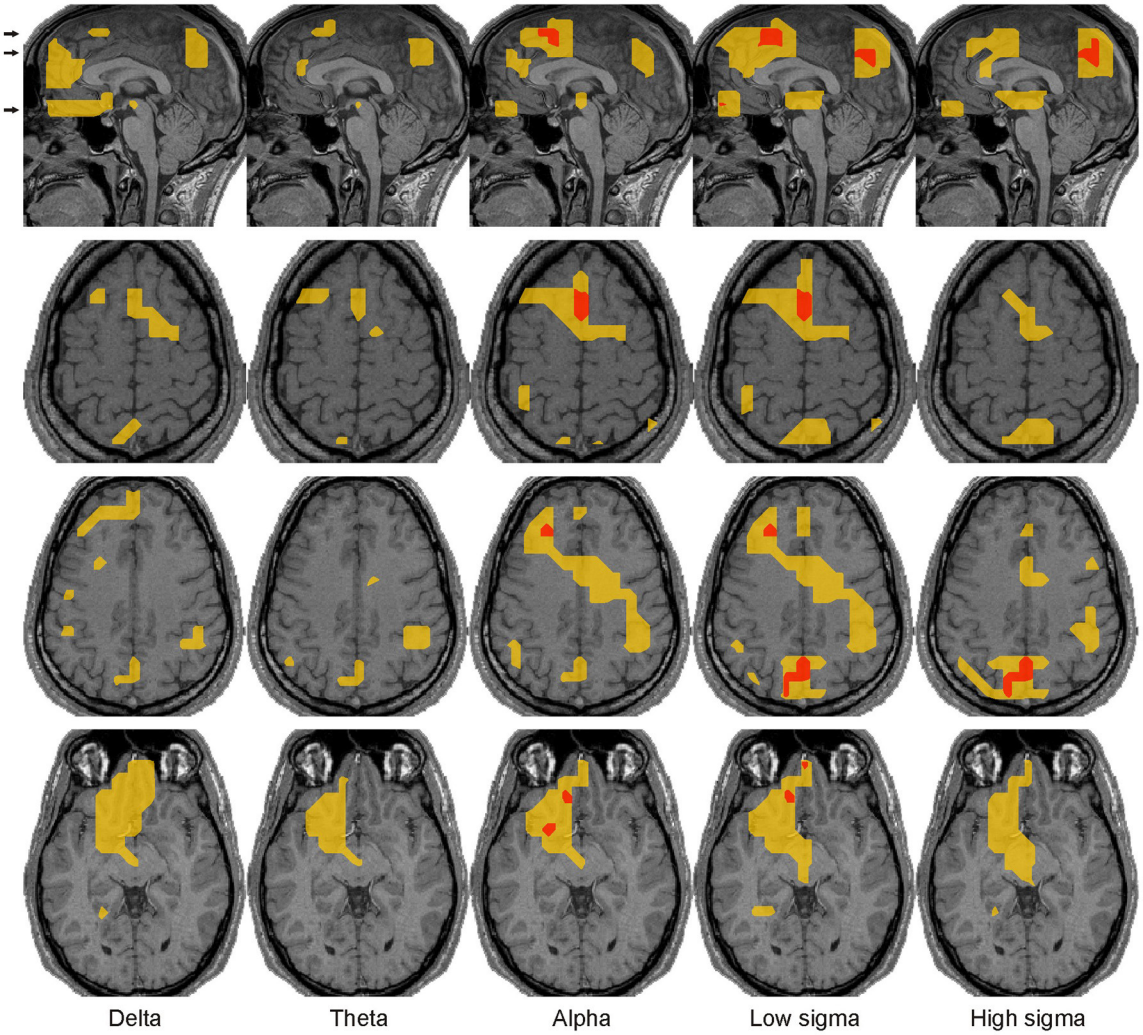
Spectral power changes during spindles. The same conventions are used as in Figure 6.

**Figure 8:**
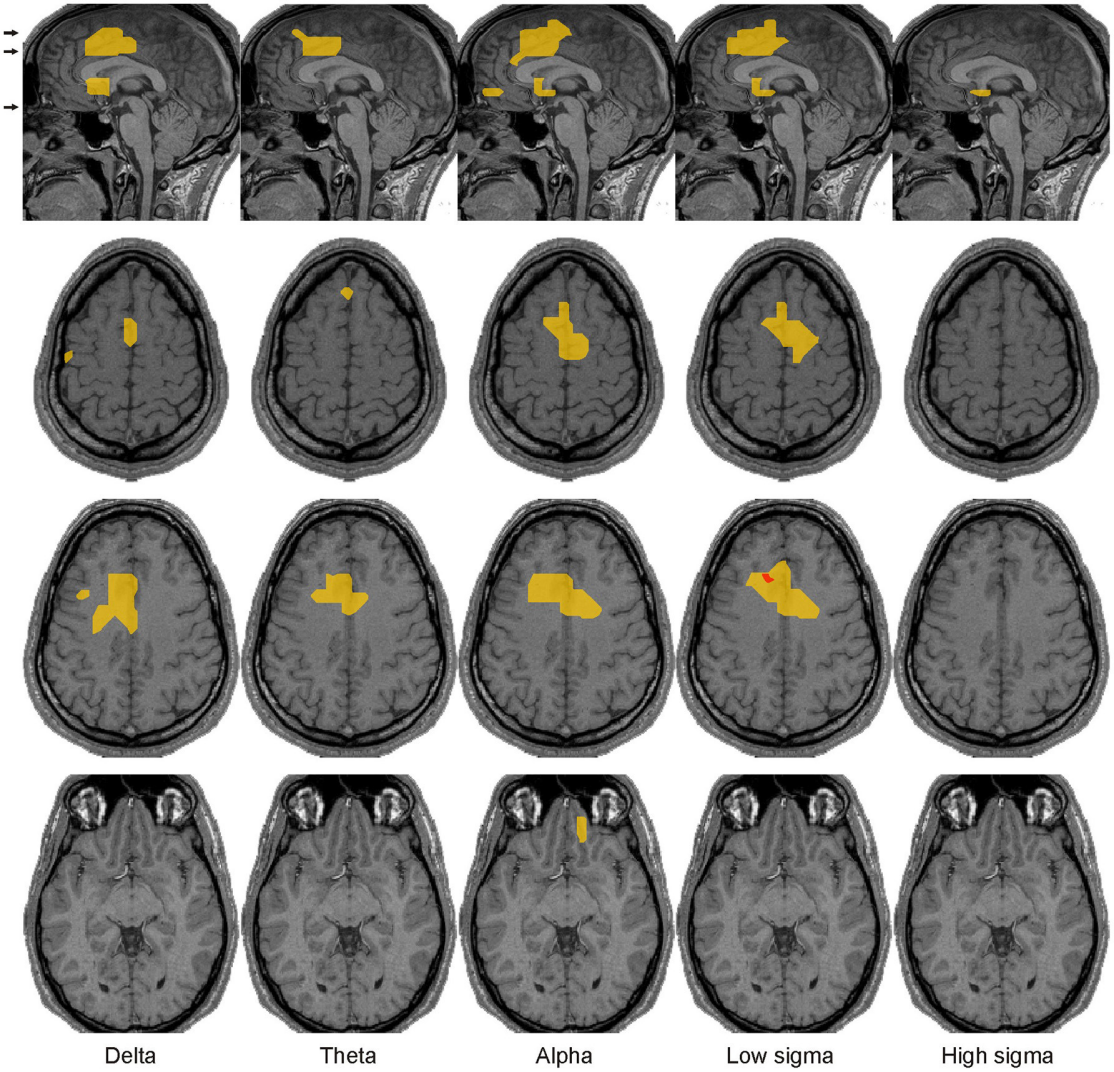
Spectral power changes before KCs. The same conventions are used as in Figure 6.

**Figure 9:**
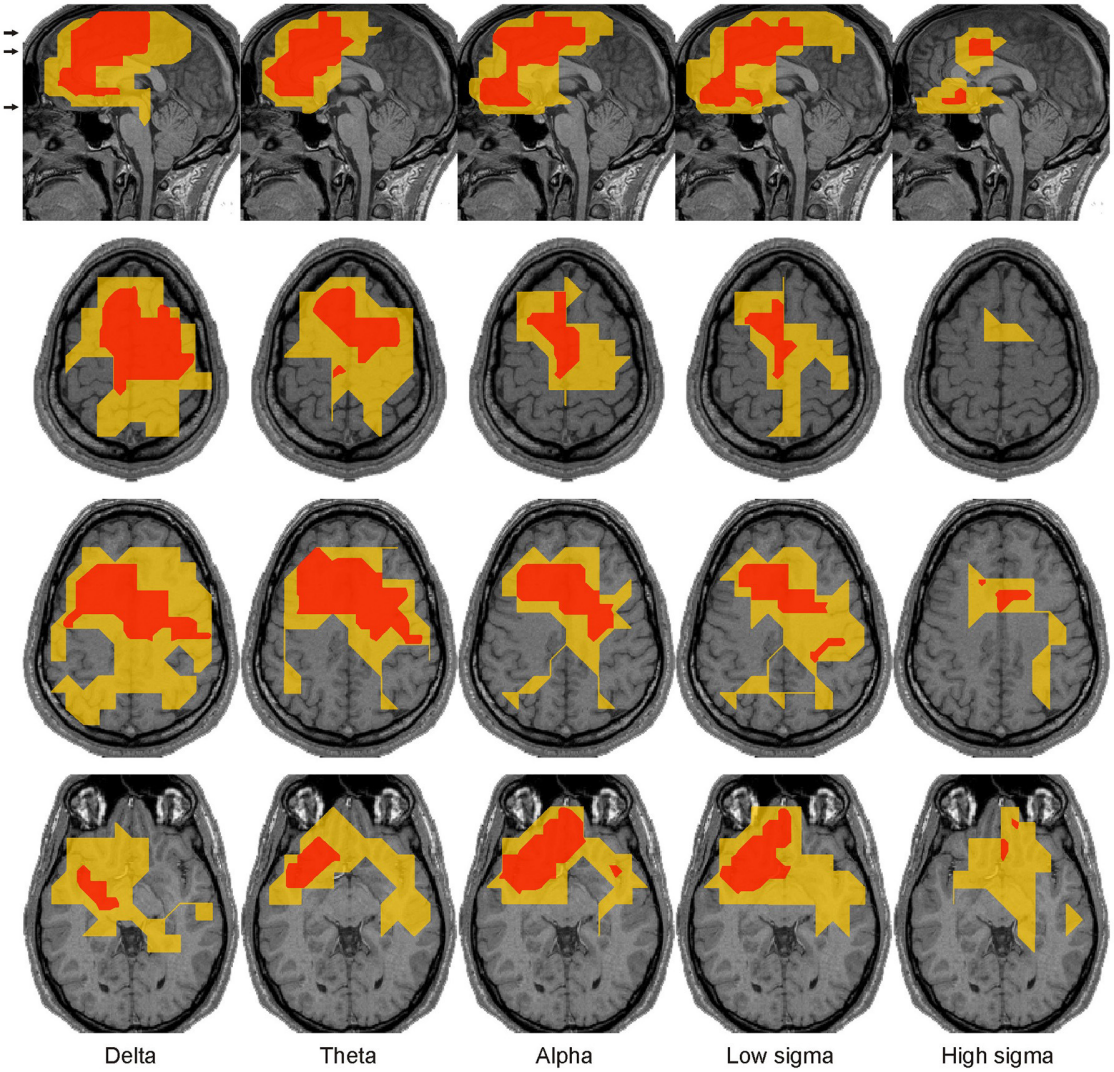
Spectral power changes during KCs. The same conventions are used as in Figure 6. In the right hemisphere prominent increases extended posteriorly within the dorsal frontal cortex toward the precentral gyrus, with a wider spread in the low than high frequencies. Prominent increases in basal forebrain were confined within the left hemisphere identified in all frequency bands from delta to high sigma, with the most widespread in the alpha and low sigma bands where they extended around OFC and NBM.

**Figure 10:**
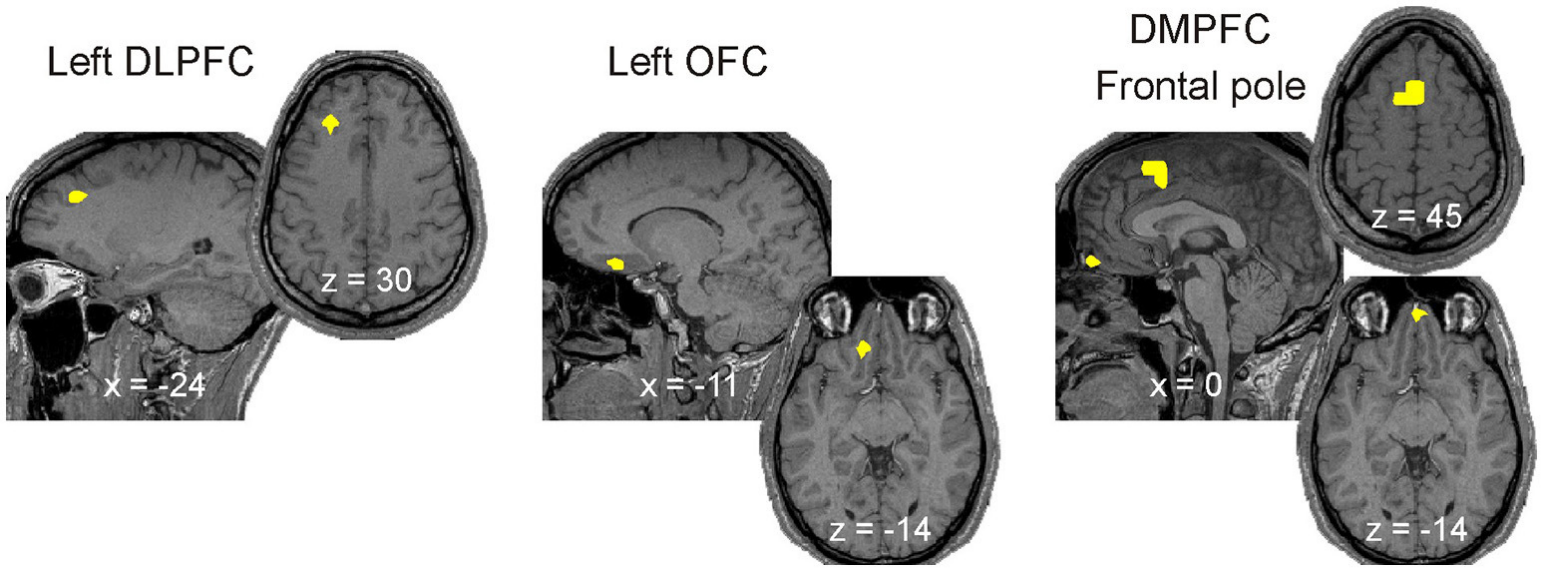
Brain activations strongly associated with the emergence of spindles and KCs, i.e., satisfying the three criteria for increases leading from awake state to spindles and KCs (see Section Continuity of Common Regional Spectral Power Changes Leading to Spindles and KCs). The four brain areas with continuous increases in activity (yellow) across key periods of sleep leading to the emergence of KCs and/or spindles in all subjects. For each case a pair of orthogonal MRI slices (sagittal and axial) is displayed. Common activity in the left DLPFC and left OFC are superimposed on left and middle pairs of MRI cuts, respectively. The activity in the DMPFC and Frontal pole regions are superimposed on the pair of MRI cuts on the right; DMPFC/Frontal pole activity is more superior/inferior on the sagittal slice and is shown on the upper/lower axial slice. The x and z coordinates of the MRI slices in the Talairach space are provided at the bottom of sagittal and axial slices, respectively. More details for each of the four ROIs can be found (a) all three Talairach coordinates in Table 1, (b) specific spectral power change in each POI and its statistical significance in Figures 4–9.

**Figure 11:**
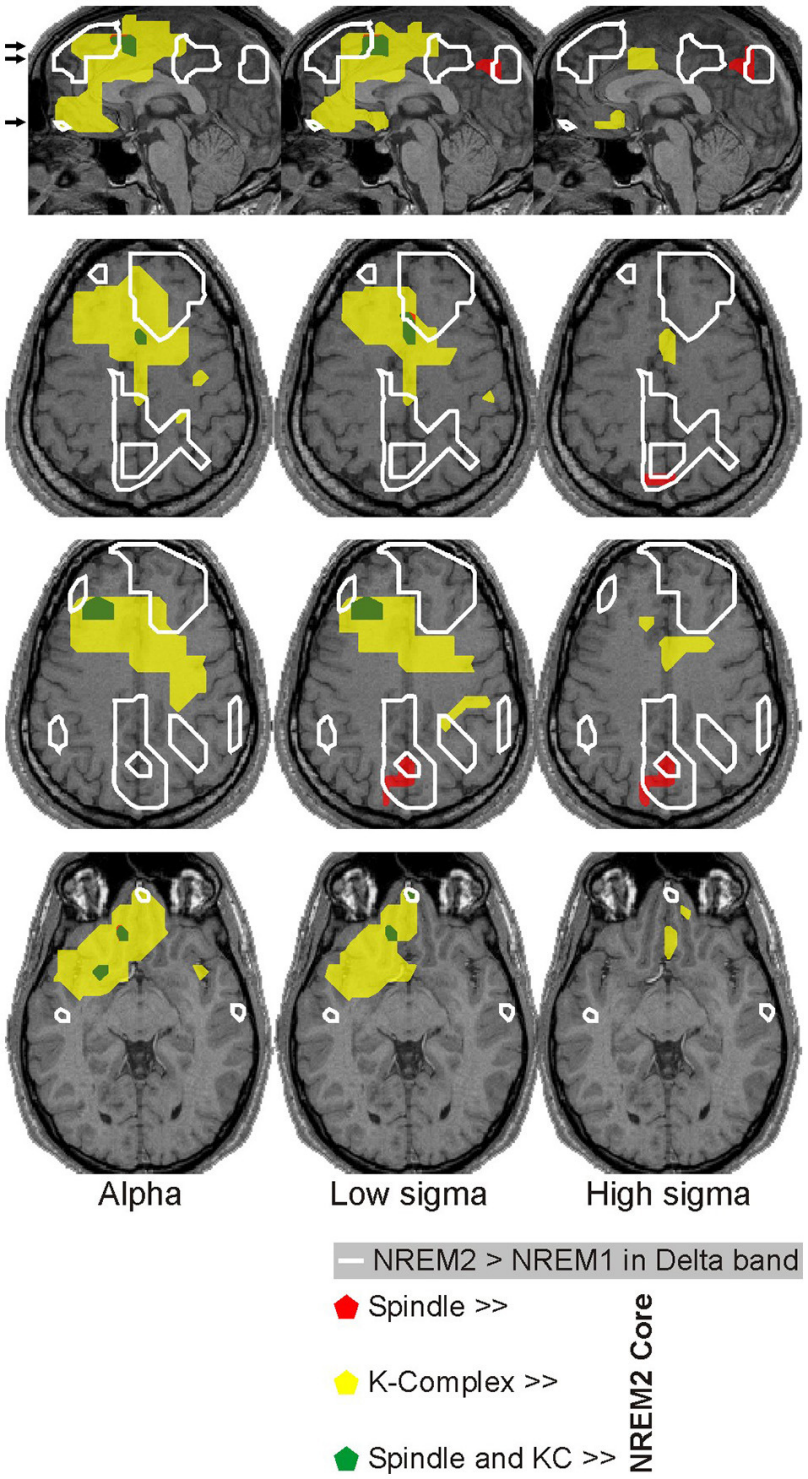
Spatial organization of spindle- and KC-related areas common across all 4 subjects. Prominent (*p* < 0.00001), increases of brain activity during spindles (red) and KCs (yellow) over NREM2 activity during core periods are shown on the midline sagittal slice and three axial slices. The positions of the axial slices are indicated by black arrows on the sagittal slice. The areas with prominent increases in activity during both spindles and KCs are shown in green. White contours encompass regions with modest (*p* < 0.05) increases in delta band activity in NREM2 over NREM1 activity during core periods (“NREM2 low frequency areas”). The same symbols as in Figure 5 are used to indicate modest and prominent changes in spectral power.

The resultant spectral statistical maps were combined across subjects to identify common changes in brain activity at pre-defined statistical thresholds. The statistical maps of each subject were first transformed into the Talairach space assigning a new *t*-value in this common space by smoothing the original *t*-values within a sphere of radius 12 mm using a Wood Saxon kernel of radius 7 and decay constant 4 mm. This operation had two effects. First it allowed for possible errors in localization (which through segment selection from periods of very small head movement should be small, within a few millimeters), individual differences in anatomy, and inherent inaccuracy of transformation into a common space. Second it smoothed and further reduced the value of the *t*-test around the peak voxels, thus ensuring that high *t*-values in the common space had also a reasonable extend. The grand sSPM results were finally computed, separately for each POI comparison by counting for each voxel the number of subjects which showed increase or decrease of activity at a pre-defined threshold of *p*-value (after Bonferoni correction for multiple voxel comparisons). No additional correction was made because the conservative nature of the Bonferoni correction for the 1,000 or so voxels would have more than compensated for the few frequency bands used in the analysis. The resulting grand sSPM results in the Talairach space were then back-transformed to the anatomical space of one subject so they could be displayed in the background of that subject's MRI. We will report only results for voxels satisfying the predefined threshold for ALL four subjects, using either a threshold of *p* = 0.05 and referring to corresponding changes as modest or for the more stringent threshold *p* = 0.00001 referring to such changes as prominent. Figures 4–**11** describe results using grand sSPM maps for different POI comparisons.

### Regions of interest and time domain analysis

Spherical regions of interest (ROI) with a radius of 1 cm were defined in one of two ways. First, we identified all prominent (*p* < 0.00001) increases in spectral power before and during spindles over NREM2 core periods from the combined-across-subjects sSPM described in Section Grand sSPM and Reporting of Statistical Significance. We transformed the common activations to the MRI of individual subjects to define for each one a second set of ROIs. Second, we used an automated procedure to identify the strongest activations at the extrema of individual spindles (positive and negative peaks and zero crossings) from the spatiotemporal brain activation maps (produced directly by MFT in the time domain). This, time domain procedure, was performed in the native space defined by the anatomy (MRI) of each subject and separately for each identified spindle. This analysis produced large number of ROIs with the same voxels identified as the strongest generator at peaks and zero crossings, but at different single waves of a spindle or different spindles.

Regional activation curves (RAC) for all ROIs and spindle epochs were then generated from spatiotemporal brain activation maps (i.e., MFT estimates) by integrating, for each time sample of 1.6 ms, the projections of the current density vectors along the principle direction in the ROI (Poghosyan and Ioannides, 2007, 2008). A RAC describes the activation time course along its dominant direction, which is calculated using circular statistics (Fisher, 1993), taking into account both magnitude and direction of the current density vectors (Ioannides et al., 2005). We studied the RACs in both the wide band and after filtering each current density component in the sigma band (11–17 Hz). The squared RAC moduli were displayed together with the spindle (EEG trace filtered in the same sigma band) to facilitate the comparison of the time courses of the EEG spindle and the regional source power in the wide and sigma bands.

## Results

A key aim of our work is to provide a comprehensive description of the changes from awake state to light sleep and the periods before and during the spindles and KCs. Therefore, in addition to the new findings from our computations we also bring together earlier findings. These findings are spread over many publications covering the many decades of research on the electrophysiological correlates of sleep, first at the level of actual measured signal (EEG and MEG) or invasive recordings in animals and more recently with a flood of recent studies with intracranial and non-invasive recordings in humans. We recognize that it is impossible to adequately cover this huge literature in the references of one manuscript and that, even if the references are given, it will be difficult for the reader to clearly separate new from old results. We have therefore added comments and some references in subsections of this, the Results Section, to make clear which of our results are novel and which are not, providing the reader with the references that are, in our opinion, most relevant.

Figure 2 shows a typical example of a night's recording from a single subject: the hypnogram of sleep stages, head movement during the sleep and the recorded electrophysiological signals (EMG, EEG, and MEG) are shown. Only the data chunks (3 min) with minimal head movement (<5 mm) were used for detailed analysis; spindles, KCs, and core periods were identified within these chunks. The raw EEG and MEG signals within each data chunk were dominated by the intermittent appearance of the characteristic large graphoelements.

### EEG and MEG signals during POI

Figure 3 shows the results of the frequency analysis at the level of MEG and EEG signals described in Section Displays of the Raw MEG Signal and Auxiliary Channels. The EEG and MEG spectra are characterized by a gradual and smooth progression from wakefulness to NREM1 and NREM2 leading to spindles and KCs. The expected reduction in the alpha band power in transition from wakefulness to light sleep (“alpha block”) is evident in core periods, especially over parietal sensors (Figure 3A). The power spectra of the core periods show also reductions in the frequencies above ~8 Hz and increases in the lower frequencies. The growth of the low frequency power continued further in the periods before and during spindles (Figure 3B) and KCs (Figure 3C).

There is little new in Figure 3, especially in its first row. The EEG changes along the anterior-posterior midline access have been reported many times and can be seen with just a few EEG electrodes (De Gennaro et al., 2001). Changes in the spectral patterns of EEG topography across sleep stages and during periods with spindles (Werth et al., 1997) and KCs (Happe et al., 2002) have also been reported before. For the purposes of the results that follow, Figure 3 serves two purposes: First, it demonstrates that at the level of raw signal, the data we used are consistent with what earlier works reported. Second, the Figure shows that there are distinct differences in the spectra in the posterior and anterior EEG and MEG sensors in the periods just before spindles and KCs that fall roughly in between the corresponding spectral patterns of the core NREM2 period and the actual periods with spindles and KCs. This is the first and very basic baseline observation of the continuity of brain activity as sleep evolves that we are after.

### Regional spectral power of core periods changes steadily with sleep onset

The results of the main analysis described in Sections Displays of the Raw MEG Signal and Auxiliary Channels, Source Reconstruction, Fourier Analysis of MFT Solutions, and Spectral Statistical Parametric Mapping (sSPM) for Each Subject are presented in terms of grand sSPM maps (described in Section Grand sSPM and Reporting of Statistical Significance) beginning with Figure 4 showing a panoramic overview of significant changes in different frequency bands, identified close to the midline in all subjects during the key POIs, superimposed on a mid-sagittal (midline) slice.

The statistical comparisons between **core periods** of light sleep and wakefulness showed widespread prominent increases mainly in low frequency spectral power (delta and theta) and prominent decreases in higher bands (alpha and sigma, Figure 5A). The low frequency increases were found in dorsal medial cortical areas, starting in NREM1 and spreading more widely in NREM2. The only prominent reduction in theta band in NREM2 was found in the dorsal brainstem [in the region of locus coeruleus (LC) nucleus; marked by a white arrow in Figure 5A]. The decreases in the alpha and sigma band power were found in ventral posterior, occipitoparietal, and sub-cortical areas (mainly dorsal brainstem).

The direct comparison of NREM2 to NREM1 core periods showed modest increases mainly in low frequencies (marked by the white contours in Figure 5A). The increases in the delta band were fairly extended in the dorsal frontal and parietal areas, hereafter referred to as “NREM2 low frequency areas,” while increases in the alpha and low sigma bands were focal in the frontal pole, rostral anterior cingulate cortex (rACC), and anterior medial prefrontal cortex (MPFC).

### Regional spectral power changes in anticipation and during spindles and KCs

Modest increases in activity from NREM2 core periods to periods before spindles were found in the delta and theta bands (Figure 5B). Medially, these increases were localized in the sub-genual anterior cingulate cortex (sgACC) and frontal pole in the delta band only, and in the rACC and dorsal MPFC (DMPFC, BA6) in both delta and theta bands. Additional modest increases, predominantly in the same low frequency bands, were found in a number of areas away from the medial brain structures, including in the left hemisphere dorsal lateral prefrontal cortex (DLPFC, BA9), orbital frontal cortex (OFC, BA11) and basal forebrain (in the anterior region of nucleus basalis of Meynert, NBM, BA25), and bilaterally around the central sulcus (Figure 6).

Prominent increases **during spindles** over NREM2 core periods were found only in the alpha and sigma bands: in the frontal pole (low sigma only), DMPFC (alpha and low sigma), and precuneus (BA7, sigma only) medially (Figure 5B) and more laterally in the left hemisphere DLPFC, OFC, superior parietal lobule (SPL, BA7) and basal forebrain (alpha only) (Figure 7). Modest activations (*p* < 0.05) were identified in a wider set of areas, including around the bilateral central sulcus, which were more widespread in the right hemisphere (Figure 7).

Modest increases in activity **before KCs** over NREM2 core periods were found mostly in medial frontal brain areas, in the region of sgACC and DMPFC in nearly all frequency bands and in the frontal pole in alpha band (Figure 5C). The few increases off the midline sagittal slice are shown in Figure 8. **During KCs** prominent increases in activity extended around the pre-KC medial frontal areas (Figure 5C). The increases covered much of the ventral and dorsal MPFC forming a medial frontal arc (mFrArc) that followed the cingulate from its anterior ventral end to its middle-dorsal aspect. The increased activity extended also laterally into both hemispheres (Figure 9).

In the mid frontal cortex, persistent increases in activity were seen over a range of frequencies in well circumscribed focal areas close but distinct for spindles and KCs and with different frequency signatures (Figure 5). For spindles, the key area was situated caudally to the DMPFC identified in our earlier work with gamma band consistently increasing through the sleep stages with highest power during REM (Ioannides et al., 2009). The increases were modest and confined to low frequencies (delta and theta) before spindles but became prominent and confined to the alpha and low sigma bands during spindles. For KCs, the key area was also rather focal situated caudally and more ventrally to the DMPFC spindle area, in the mid cingulate cortex (MCC). The increase in this area was present both before and during KCs, modest and more circumscribed before KC and prominent and widespread during KCs. There was no clear cut separation between the frequency ranges before and during KCs: increases were identified both before and during KCs from delta to low sigma. The extensive dorsal prominent increase in activity during KCs in the low frequencies (delta to low sigma) was confined in the high sigma band to circumscribe increases in the MCC and sgACC areas (Figure 5C).

Bits and pieces of the information displayed in Figures 4–7 can be found spread in many recent studies, notably using LORETA with EEG (Anderer et al., 2001), MEG (Dehghani et al., 2010a), fMRI (Schabus et al., 2007; Caporro et al., 2012), and intracranial recordings (Andrillon et al., 2011; Staresina et al., 2015). However, these earlier reports do not contain either the spatiotemporal detail or the comprehensive and principled way specific regional spectral properties vary from awake state to core periods of NREM1 to NREM2 and from the core period of NREM2 to the periods before and the periods during spindles and KCs. Some of our results simply add detail to what is already known, e.g., the distinction of slow and fast spindles and their distribution in the brain. The details however in the fine responses of key areas are all important together with the distinct role these areas play in the awake state, e.g., in monitoring the environment or enhancing the response to salient stimuli. The combination of the detail provided by our analysis and the knowledge of what the identified areas do in the awake state allowed us to put together a plausible explanation of the role of each of these areas and the significance of their concerted activation or inhibition in each POI, fully consistent with the emerging consensus about the relation of sleep with memory consolidation. The detailed analysis of the regional spectral properties throughout the brain and separately for each POI, allowed for the first time, the identification of a small number of areas that through their continuous changes in spectral pattern of activity across POIs seem to play a controlling role in the events of light sleep, as we describe in the next subsection.

### Continuity of common regional spectral power changes leading to spindles and KCs

We defined three selection criteria for identifying the brain regions where activity continuously increased across key periods of light sleep leading to the emergence of spindles or KCs: independent of the frequency band(s) spectral power had to increase (1) from wakefulness to NREM2 core periods, (2) from NREM2 core periods to periods before the graphoelement (spindle or KC), and (3) from NREM2 core periods to periods during the graphoelement (spindle or KC). Only three focal brain regions were found to satisfy these five criteria (one criterion for core states and two for spindles and two for KCs) left DLPFC, DMPFC, and frontal pole. One additional region, left OFC (anterior to NBM) satisfied the criteria only for spindles (Figure 10); this region showed no increase in the spectral power in any band before KCs (see the bottom row of the Figure 8). The common increases shown on Figure 10 are at different frequency bands in the different POIs, specifically: each area shows prominent increases in spectral power from wakefulness to NREM2 core periods in the delta and theta bands, modest increases from NREM2 core periods to periods before both the spindles and KCs and prominent increases during both the spindles and KCs compared to NREM2 core periods.

### Spatial relationship of spectral power changes

Figure 11 shows the “NREM2 low frequency areas” (white outline), i.e., the areas where delta band activity increased from NREM1 to NREM2 core periods, together with the areas of increased alpha and sigma band power from NREM2 core periods to the periods during spindles and KCs. The display demonstrates that the three frontal areas (shown in green) that are prominently increasing in the sigma band for both spindles and KCs (i.e., DMPFC, frontal pole, and left DLPFC) are consistently located at the edges of the “NREM2 low frequency areas.” Similarly, nearly all brain areas showing prominent increase in the higher frequencies (alpha and sigma bands shown in red) during spindles were found at the edges of the same “NREM2 low frequency areas,” including the only area where prominent sigma band activity was identified during spindles, but not during KCs, in the posterior parietal cortex (last image in the top three rows of Figure 11). Furthermore, the more widespread KC-related areas (shown in yellow) filled largely the space between the different “NREM2 low frequency areas.”

### Temporal dynamics of brain activity during spindles

Comparing results from different studies using different modalities or even from the same modality but employing different statistical comparisons (e.g., different baselines) requires paying special attention to details. In general complex and distributed sources will show differently when different modalities (EEG, MEG, intracranial recordings, fMRI etc.) are used. Mechanisms underlying the differences may arise from physiology (neuronal networks) as well as physics (differences in sensitivity as prescribed by the differences in the lead fields for EEG and MEG). Specifically in relation to spindles, recent studies found differences in power and temporal profiles for the spindles identified by MEG and EEG. Also these studies reported that MEG spindles tended to be less coherent (Dehghani et al., 2010b) and less correlated throughout the cortical surface (Dehghani et al., 2010a). The lack of correlation was also reported in studies of local spindles using intracranial recordings (Andrillon et al., 2011; Frauscher et al., 2015). One possible contribution to the differences may be related to MEG only representing intracellular currents from action potentials back propagating into apical dendrites in those instances when individual spindle depolarizations exceed threshold. A second possibility considers the difference in what each modality preferentially sees—MEG seeing only the tangential diploes in sulci, where synchronous dipoles on both sides of a sulcus may be expected to cancel each other. The observed differentiation led some authors to propose that MEG and EEG spindles are generated by different thalamic projections (Bonjean et al., 2012). However, 85% of all detected EEG spindles were also seen in the MEG (Dehghani et al., 2011a) and MEG spindle power increased drastically in the presence of detected EEG spindles, suggesting (as one would expect from physics) that the signals in both recording modalities result from the same neurophysiological generators (Klinzing et al., 2016). It is important to emphasize the plural in the last statement; spindles appear to represent a mixture of different types of phenomena—some exquisitely focal and others more broadly spread, depending on the degree to which thalamic pathways, core or matrix, contribute to their generation; the EEG more likely representing spindle types generated by matrix (nonspecific) thalamic nuclei, and MEG representing spindles generated by core (specific) thalamic pathways (Bonjean et al., 2012; Piantoni et al., 2016, 2017). We discuss the relation between our results and the results of key earlier studies in detail in the Discussion session.

In the remainder of this subsection we address an apparent paradox related to the results derived from the spectral comparisons between conditions and/or POIs and presented in Figures 5–11. These results are in broad agreement with results from fMRI, some EEG studies, notably (Anderer et al., 2001), and intracranial recordings (for a detailed discussion see Section Relationship to Earlier Studies) but, they do not include the areas identified by other studies where the generators were localized at the peaks of time series of spindle activity in the EEG and MEG signal (Manshanden et al., 2002; Ishii et al., 2003; Urakami, 2008; Gumenyuk et al., 2009). To resolve this paradox we compute the time courses of regional activations from the MFT solutions for the key areas identified in the analysis of spectral properties and for areas which stood out clearly at the positive and negative peaks of EEG spindles.

The in-depth time-domain analysis of the brain activation maps during spindles revealed strong activity in parietal, central, and frontal areas around the central sulcus, fully in line with a series of recent studies using EEG/MEG and/or intracranial recordings (Manshanden et al., 2002; Ishii et al., 2003; Urakami, 2008; Gumenyuk et al., 2009; Dehghani et al., 2010a; Frauscher et al., 2015; Staresina et al., 2015). The strongly activated areas, which stood out clearly at the peaks (positive and negative) of EEG spindles, were not the same as the areas that appeared as prominently in the spectral analyses (Figures 5–11).

To investigate the temporal dynamics of regional brain activity, we identified all the areas that were consistently and strongly activated both at the peaks of spindles and/or the sSPMs. We generated RACs for each one of these areas to study the spatiotemporal evolution of activity during the spindle periods as described in Section Fourier Analysis of MFT Solutions (Poghosyan and Ioannides, 2007, 2008). The EEG and MEG signals and RACs were studied for each subject at different temporal windows, ranging from a few milliseconds to the full 3-min data chunks. The results described in the next two figures, are typical of what we found in the spindles of each of the four subjects we studied. For ease of reference key ROIs that give focal activations in the sSPMs and during spindles are collected together in Table 1 where their Talairach coordinates and the figures where they appear are provided.

**Figure 12:**
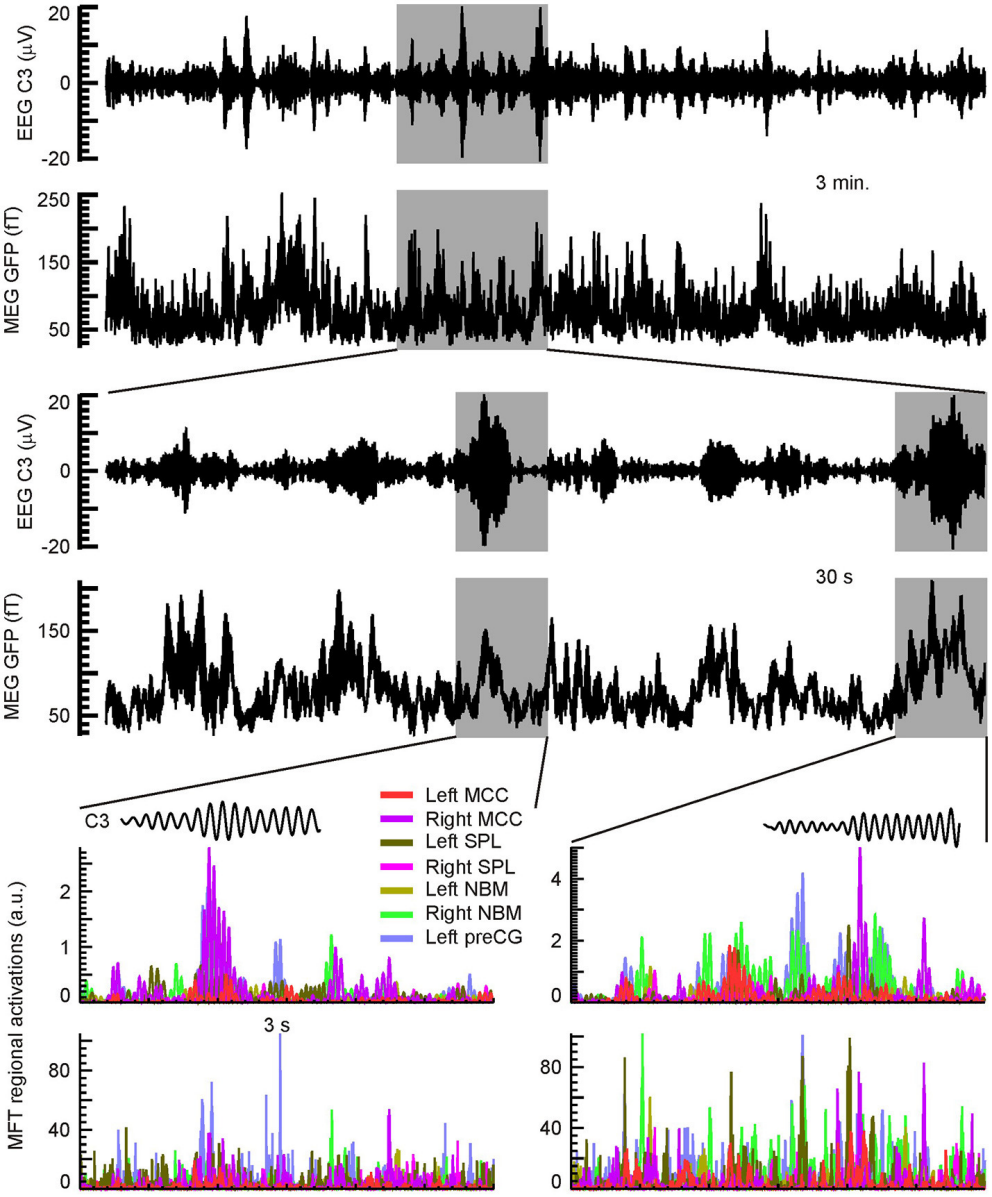
Typical example of a hierarchy of time courses involving spindles. The top pair of rows shows the EEG (C3) and MEG (GFP) during a 3 min run; the EEG and raw MEG signals were both filtered in the sigma band (11–17 Hz). The next two rows show the same signals for the 30 s shaded segment, where five spindles can be identified. The last two rows show the RACs of 7 ROIs, during the two most prominent spindles in the 30-s segment (shaded for highlight in the middle pair of rows). See Table 1 for details on ROIs. RACs were squared to emphasize the temporal locations and sequence of the peaks. The RACs presented in the penultimate row were filtered in the sigma band before squaring. The insets just above the RAC plots show the concurrently recorded EEG (C3) spindles (11–17 Hz). Note the difference of nearly two orders of magnitude between the filtered (penultimate row) and wideband (last row) RACs.

The top pair of rows in Figure 12 shows typical EEG (C3 electrode) and MEG (GFP) signals from a 3-min long NREM2 sleep segment with multiple spindles. The second pair of rows zooms on the 30-s segment shaded in the first two rows. In this 30-s period at least 5 spindles can be seen, and the two prominent ones are shaded for highlight. In the last two rows the RACs of 7 ROIs are over plotted together zooming on the two highlighted spindles of the 30 s segment above, showing the power in each ROI first confined in the sigma band (penultimate row) and finally in the wide band (lower row).

**Figure 13:**
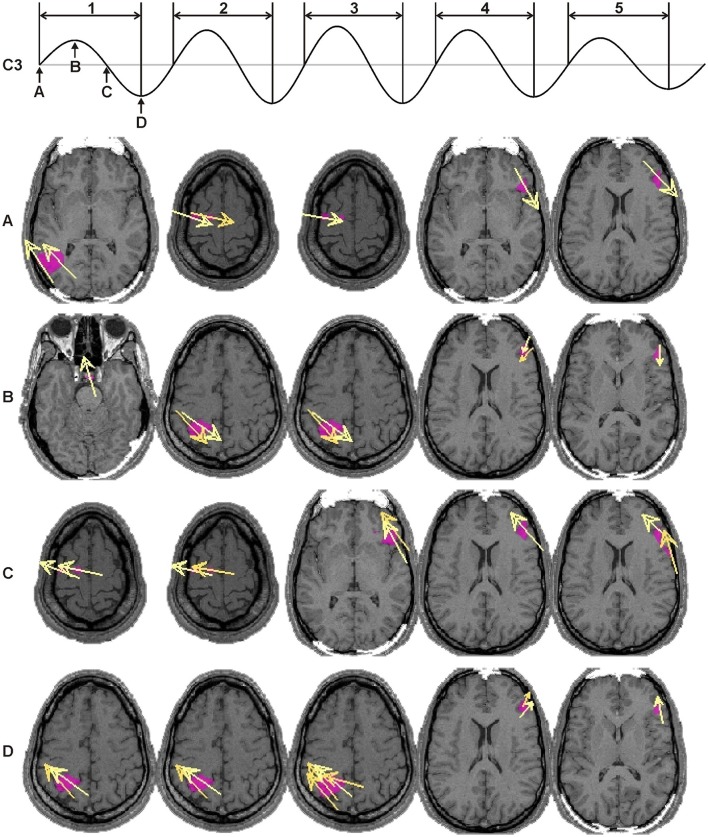
Typical example of a sequence of dominant activations in a single spindle. The top row shows an EEG (C3) spindle (11–17 Hz) with five cycles. Four characteristic points are defined for each cycle: positive zero crossing **(A)**, positive peak **(B)**, negative zero crossing **(C)**, and negative peak **(D)**; in the figure they are marked on the first spindle cycle. Below, the dominant brain activations are shown for each spindle cycle (columns) and characteristic point (rows). Axial slices with the strongest activity are shown with a pink blob marking the area with modulus value above 90% of the instantaneous maximum and yellow arrows show the direction of the current density vectors.

Figure 13 shows a typical example of the sequence of strong brain activations in the sigma band during one spindle. The activation of any one strong generator is brief, it lasts at most few peaks or zero crossings of a spindle. Referring back to the last two rows of the Figure 12 it is clear that what is seen in these maps is a small part of the overall signal (compare the scales in rows 5 and 6 of Figure 12) and that the duration of individual activations is even shorter than what is seen in the displays of Figure 13 if the unfiltered activations are displayed.

In summary, the analysis of brain sources at the peaks (positive and negative) and zero crossings of EEG spindles reconciles all observations. The observed patterns are as described in studies using intracranial recordings: each EEG/MEG recorded spindle emerges from a transient overlap of asynchronous activity of distinct focal cortical sources rather than widespread synchronous oscillations. The analysis also resolves the paradox it set out to resolve by identifying in addition to the sources that were identified in the analysis of regional spectral changes between conditions the areas identified in earlier studies focusing on the EEG and MEG peaks of activity (Manshanden et al., 2002; Ishii et al., 2003; Urakami, 2008; Gumenyuk et al., 2009). The differences simply arise from the different way the data were analyzed: the spectral comparisons are based on Fourier analysis, so the emphasis is on rhythmic activity across a wide period of time while the time domain analysis focuses on the instantaneous patterns of activity at each time point that require either a wide frequency range to identify the absolute peaks in power or using narrow band filters to identify the time points of similar phases (peaks or zero crossings) within the selected narrow frequency band. There is no reason why the corresponding generators should coincide, in fact uncertainty principle warns us that we should not expect it. A wavelet analysis is of course an intermediate approach that could be used to explore more deeply the underlying phenomena and specifically to investigate in detail the way the sources in the two different sets are related through cross frequency coupling in amplitude and/or phase. This analysis is beyond the scope of this manuscript.

## Discussion

### Overview of the results

Tomographic data from whole night sleep MEG recordings were analyzed by two complementary approaches focusing, respectively on the **frequency** and **time** domains. With the first (frequency domain approach) we examined the changes in regional spectral power changes from wakefulness to light sleep (NREM1 and NREM2) leading to the generation of spindles and KCs. We focused on sleep periods without any obvious graphoelements (representative of the core baseline activity of each sleep stage), and periods immediately before and during the characteristic NREM2 graphoelements (spindles and KCs). Activity was observed to steadily increase or decrease depending on the frequency band and brain area in a consistent manner, from wakefulness to the core periods of light sleep. The transition from core periods to the periods immediately before and those during both, the spindles and KCs contained only increases in spectral power. With the second (time domain) approach, we studied the tomographic activity estimates across time through sleep stages and within NREM2, with emphasis on spindles at distinct temporal scales ranging from a few milliseconds to many minutes.

### Frequency domain analysis

The analysis of the raw signal (Figure 3) is consistent with previous reports of increased EEG delta and theta and decreased occipital alpha and higher frequencies at sleep onset (Alloway et al., 1999). The results of the spectral analysis of the tomographic solutions (Figures 4–11) provide precise spatial and spectral specificity to the hints of Figure 3. Reductions were observed only in ventral posterior cortical and subcortical areas in alpha and sigma bands (two left columns in Figure 4), confirming the well-known “alpha block” at sleep onset observed mainly in occipital sensors. Our results provide also a wider picture: the reduction in alpha activity affects a wider network, extending into ventral areas and hence may guide the search for subcortical areas producing alpha in wakefulness as suggested for the pedunculopontine nucleus (Androulidakis et al., 2008).

Spindles and KCs emerge most often in close time proximity. Three focal frontal areas were found to be strongly associated with the emergence of both spindles and KCs: the DMPFC, frontal pole and left DLPFC (Figure 10). Activity in these areas steadily increased from wakefulness to NREM2 core periods, then from NREM2 core periods to periods before spindles and KCs in low frequency bands and in the alpha and sigma bands during spindles (and KCs). These three areas are displayed in Figure 10 in sagittal and axial views, together with a fourth area OFC, the only other area that satisfied all the continuity criteria but only for spindles.

The seeds for the differentiations leading to spindles (mainly dorsally) and KCs (mainly ventrally) are spectral changes in specific sites from NREM1 to NREM2: increase dorsally (DMPFC and precuneus) for delta and theta as well as ventrally (sgACC and frontal pole) for alpha and low sigma. KCs are characterized by further expansion of the activations observed since sleep onset in both dorsal medial frontal cortex and most strikingly in the mFrArc for all frequency bands. In the alpha and low sigma band the midline prominent increases in activity are focal in the DMPFC for spindles. For KCs the increases are more widespread in spatial extend and in the spectral content. Spatially they extend from the spindle area in a caudal and ventral direction spreading over the midline part of the MCC and the adjacent left and right parts of the cingulate. In the high sigma band the prominent increases in activity completely separates for spindles and KCs: for spindles prominent increases are identified in a new area, the posterior area (SPL) (for both low and high sigma), while for KCs the prominent increases in the high sigma band are confined to the MCC, i.e., more the ventral and medial part of wider increases seen in the lower frequencies. The activation of rACC also differs for spindles and KCs, something that might relate to the alerting/environment monitoring function of this area (Bush et al., 2000; Luu and Pederson, 2004; Rudebeck et al., 2014).

### Relationship to earlier studies

Our spectral analysis confirms the distinction between “slow” frontal and “fast” parietal spindles (Anderer et al., 2001; Andrillon et al., 2011; Dehghani et al., 2011b) as well as the widely distributed, but mainly mid-frontal origin of KCs (Colrain, 2005; Cash et al., 2009). In addition, our results provide a spatiotemporal qualification in terms of increases over the baseline NREM2 core activity and about the frequency content of the generators (see Figures 4, 5). Importantly the frontal low sigma spectral increase is now seen to be the upper end of spectral changes in a wider band that extends down to the theta range. The increases in spectral power in the theta to low sigma range are also distributed more widely across the frontal and posterior brain areas while the high sigma increases are confined to the SPL areas in the posterior parietal cortex. These findings explain why it is easier to reliably detect fast spindles with any one of a range of algorithms while for slow spindles more sophisticated algorithms are needed primed to individual frequencies (Ujma et al., 2015) and possibly spindles at very different locations.

Our identification of increase in delta activity in frontal areas during the pre-spindle period fits well with recent intracranial recordings demonstrating a functional coupling of slow oscillations and sleep spindles (Staresina et al., 2015). In the Staresina study it was further demonstrated that time hierarchy of oscillations continues with hippocampal ripples (fast oscillations ~200 Hz) clustering in the troughs of spindles, providing fine-tuned temporal frames for the hypothesized transfer of hippocampal memory traces.

We find similar, but not identical midline frontal and posterior parietal areas involved in the progression from wakefulness to NREM1 and NREM2 and in the periods before and during spindles as the areas identified as steadily increasing in the gamma band in deep sleep and REM (Ioannides et al., 2009). See the pairs of DMPFC and left SPL ROIs in Table 1. All the areas are in the left hemisphere, they are close to the midline and they are at the center of areas associated with the default mode and Theory of Mind networks. The two new areas identified here in the anterior frontal and posterior parietal dorsal midline areas are involved in spindle generation that could be associated with memory consolidation (Rosanova and Ulrich, 2005). The activities identified in the two studies show that these midline areas play important roles in much of sleep (at least stages NREM2 and REM) with a possible role the maintenance of a stable representation of self and its history that is integrated and consolidated with the ever changing external environment.

We note the relevance of our findings to a series of recent studies of the most prominent activation in the entire ACC (BA 24), pregenual ACC (BA 33), and especially sgACC (BA 25) at times preceding both spindles and KCs—distinguishing them from NREM2 background—and further expanding only during KCs (Figures 4, 5). We point out first that the demonstrated activations of midline frontal areas are in agreement with studies on evoked KCs (Laurino et al., 2014) and the implication of sgACC in sleep based on the observation that neurons in primate sgACC start firing at high frequency right after sleep onset (Rolls et al., 2003). The ACC has been shown to play a crucial role in processing both top-down and bottom-up stimuli and in error detection (Bush et al., 2000; Allman et al., 2001). The ACC ventral part is considered to sustain autonomic arousal (Rudebeck et al., 2014). The demonstrated activations of these medial frontal areas, in consistency with the studies on evoked KCs (Laurino et al., 2014), may therefore be proposed as the anatomical basis for cognitive functions of KCs during sleep (Ramakrishnan et al., 2012). The MCC area that is most persistent during KCs, together with the left and right MCC areas nearby, correspond to the area labeled as “dorso-caudal anterior cingulate” in a recent study with 269 epilepsy patients with implanted electrodes for recording and stimulation (Voysey et al., 2015). It was found that only in this dorso-caudal anterior cingulate area, stimulation while the patients were awake lead to responses that were highly correlated with KCs occurring during sleep for each one of the 6 patients that had electrodes implanted in this area. Our study provides an integrated view of the evolution of activity highlighting precise spectral signatures that characterize in a principled way the transition from awake state to NREM1 and NREM2 core states and a clear branching in the few seconds preceding spindles and KCs, culminating in the wild excursions that characterize these two hallmarks of NREM2. In perfect agreement with the findings of combined EEG—fMRI experiments (Caporro et al., 2012; Dang-Vu, 2012), we find only increases in spectral power associated with both spindles and KCs compared with the activity in the core state of NREM2 sleep stage.

It is difficult to push the comparison between our results and the results of hemodynamic methods too far because the relationship between hemodynamic and electrophysiological activity is not yet well understood. At a general level, our sSPM results are consistent with many of the findings listed in the previous paragraph, while adding specificity with respect to the spectral content and their emergence at specific brain areas and at specific periods (core, pre- and during spindles, and KCs). This specificity allows an independent examination. We interpret the extended activations as the results of modulatory influences from specific, mainly midline structures that appear in our analysis as focal changes: changes in these control areas in the delta band (when they are inhibited) or in higher frequency bands (when they are excited). When these changes are put together and the literature about the role of these areas under different task conditions is reviewed a picture emerges that shows the spindles and KCs to be (a) the “end products” of competitive tendencies to continue with sleep and/or wake to deal with unresolved external influences and (b) an internal cognitive process that has all the hallmarks expected from the association of spindles with memory consolidation (see Section The Gating and Memory Roles of Spindles and KCs for details). The present analysis (together with our earlier two sleep studies) also demonstrates that the same MEG data when analyzed in the time domain with emphasis on the peaks of the signal, or as in our case at the extrema of the scalp EEG spindles, identifies generators at the same areas as earlier dipole analysis of EEG and MEG data (Urakami, 2008). Results from a number of recent studies and the time domain analysis reported here show spindles (and KCs) as asynchronous and apparently rather random excursions of brain activity. Viewed however in the context of the sSPM results these two hallmarks of light sleep are seen as the “end products” of a long sequence of events that preceded them since sleep onset.

### Underlying mechanisms

#### Local and global spindles

Since delta and alpha rhythms are, respectively, characterizing sleep and wake off-duty or microarousals in sleep (Halasz and Bodizs, 2013), the modest focal rACC increase in alpha and low sigma spectral power during NREM2 compared to NREM1 core period can be interpreted as an increase in monitoring during NREM2 (compared to NREM1): the initial suppression of frontal areas with sleep onset by a blanket increase in low frequencies, may be refined in NREM2 core periods with an increase in areas like the rACC that allow some monitoring of the environment. In the pre-spindle period the rACC spectral power is increased relative to NREM2 core period in the delta and theta bands, an indication of active inhibition (in an operational sense) of environmental monitoring ahead of spindle activity.

From their first description (Loomis et al., 1935) till relatively recently (Contreras et al., 1997), spindles were considered by most to be **synchronous, global** events. However, as early as the late 1960s, among the global barbiturate spindles some local ones were described with differing frequency even at loci as little as 1 mm apart (Andersen et al., 1967). Our detailed examination of the brain dynamics during spindles revealed asynchronous and diverse **focal** cortical sources, not widespread synchronous oscillations. Local spindles show no substantial synchrony to spindles in other brain loci or to epicranial spindles (Figure 12). Even within a spindle, individual wave peaks appear to maximize in different brain areas (Figure 13), which do not overlap with the areas most prominently shown in the spectral analysis. The present demonstration of an increase in MEG delta waves (Figure 3) supports the role of slow waves in light sleep, as a prime mover of NREM sleep; but the picture emerging from the spectral analysis of brain activity is far richer: prominent increases and decreases in specific frequency bands mark the progression of the background (core) activity (Figure 5A), while just prior to spindles there is an accentuation of delta band power in the medial prefrontal arc (Figure 5B). Slow oscillation may occur **locally** or more or less synchronized across brain areas depending on state of arousal (McCormick et al., 2015). Their grouping action is area-, time-, and function-dependent and may accordingly dictate the degree of synchrony among local spindles and the occasional emergence of globally synchronous ones. It is tempting to speculate that the local and global spindles serve primarily—but not exclusively—the memory consolidation and gating/hypnagogic roles, respectively. One of possible synchronizing, mechanisms is the rebound from the down state underlying the negative wave of a KC (Cash et al., 2009) or other slow frequency band activations shown here to appear just prior to spindles (Figure 5). This rebound is both synchronizing in space and depolarizing individual neurons. The latter effect may also explain part of the differentiation between more fragmented MEG and more continuous EEG records of spindles (Figure 13), i.e., EEG spindles may be generated by an entire sequence of mostly subthreshold excitatory postsynaptic potentials, while MEG could mainly reflect the action potentials occasionally produced whenever a rebound depolarization is added to the sub-threshold potentials.

Our time-domain analysis (Figures 12, 13) resolved the apparent discrepancy between the areas identified as generators of spindles by our spectral analysis and the areas identified by many earlier EEG and MEG studies focusing on the peaks of activity in the time domain (Manshanden et al., 2002; Ishii et al., 2003; Urakami, 2008; Gumenyuk et al., 2009). The stronger generators during spindles are indeed the ones identified by earlier studies in frontal, temporal, and parietal lobes, with the most common and prominent contributions from around the central sulcus. However, at these locations a strong signal is generated at all times and not just during spindles.

#### The edge effect

In general, spindles never occur during KC's negative phase, but most often emerge right after it (Kokkinos and Kostopoulos, 2011), possibly due to a rebound from a down state (Cash et al., 2009). Here spindles are associated with activations at the edges of areas exhibiting sustained increases in delta band activity during the core periods (Figure 11). We therefore suggest that in addition to the known thalamo-cortical mechanisms of spindles generation (De Gennaro and Ferrara, 2003; Llinás and Steriade, 2006), there may also exist a spindle-promoting mechanism based on an extended, spatiotemporal, **“edge effect”** (Llinás et al., 2005): the delta band low frequency oscillations will reduce the lateral inhibition, promoting coherent spindles in neighboring cortical areas (due to disinhibition of neighboring thalamo-cortical sectors).

#### The gating and memory roles of spindles and KCs

Sleep onset and sleep stage evolution has been associated to ascending influence by specific modulatory systems with both **aminergic and cholinergic systems** being deactivated in NREM (see (Lelkes et al., 2013) and references therein). Our results show decreases in relatively high frequencies in brainstem and increases in slow wave activity in frontal cortical areas (first row of Figures 4, 5A), consistent with established view of sleep onset resulting from inactivation of brainstem reticular activating system (Brown et al., 2012; Saper and Sehgal, 2013). The only focal change in activity is the prominent theta band decrease at or just above the LC for NREM2 core compared to the wakefulness. This is consistent with the well-known reduction of LC activity with sleep onset (Aston-Jones and Bloom, 1981). We observed no prominent, or even modest changes in spectral power in the brainstem and cerebellum for the periods before or during spindles or KCs relative to the NREM2 core period (Figure 4, Figures 5B,C and Figures 6–9). This almost global inactivation during the NREM-1-2 core periods may underlie the loss of consciousness upon sleep onset. The progression from NREM1 to NREM2 is characterized by focal alpha and low sigma activations in rACC (white outlines in Figure 5A) suggesting a reappearance of (some unconscious) monitoring of environmental information (Bush et al., 2000; Luu and Pederson, 2004).

The period before spindles is characterized by focal modest increases of delta and theta power in the rACC (red blobs in Figure 5B), possibly related to cancelation of the activation of this area observed in the NREM2 over NREM1 core periods, thus suspending the monitoring of the external environment. During the same period the power increases in the left side of the basal forebrain in the theta, alpha, and low sigma frequencies, more focused around the left basal forebrain for the alpha and low sigma bands (last row of Figure 6). Motivated by recent evidence suggesting that basal forebrain activation enhances cortical processing of external stimuli (Goard and Dan, 2009; Pinto et al., 2013) we propose that the alpha and low sigma increases we identified before spindles may relate to the preparations for ongoing memory consolidation. Given also the alerting role of basal forebrain (Han et al., 2014; Irmak and de Lecea, 2014) the increased alpha and low sigma spectral power in this area (Figure 6) just before spindles and its extension over the left hippocampus and thalamus during spindles (Figure 7) could be contributing to sharpening the information content of neural sequences retrieved from the hippocampus (Clemens et al., 2011), while at the same time preventing interference from external input (suppression of rACC). The outcome of this process is the accurate encoding through the prominent increases in the MPFC (alpha and low sigma) and the posterior parietal areas (low and high sigma) during spindles (yellow blobs in Figure 5B). In this picture the strong activations at the peaks of spindles may be the end of the process of imprinting the memory engram; there was no training before our experiments, so we cannot carry this line of thinking further and the localization of the activity at the extrema of spindles would appear to be rather random as it might depend on the precise content of the unknown memory to be encoded. New experiments are needed with selective training before sleep and measurements of the performance the next day to test the validity of the ideas expressed above and explore this line of reasoning to its logical conclusion.

In contrast to the pre-spindle period, the changes in the pre- and during KC periods are less discretely organized in terms of spatial distribution with no clear demarcation in the frequency bands. They appear as more generalized responses to arousal events, may be as a final attempt to annul it and maintain sleep (Halasz and Bodizs, 2013). If this is indeed the case, the interruption of spindles by KCs (Kokkinos and Kostopoulos, 2011) may be necessary so that the already growing influence of some random arousal does not contaminate the memory consolidation.

### Functional relationships of spindles and KCs

The results of our frequency analysis (described in detail in Section Frequency Domain Analysis) suggest that spindles are controlled relatively more by internal sleep state processes and contribute to synaptic plasticity, while KCs are “reactive events” (Halasz and Bodizs, 2013). Our results seen in the context of the inhibitory nature of slow waves (Cash et al., 2009) help us understand why spindles are always interrupted during the negative phase of a KC and why they are elicited with higher spectral frequency most of the time (~68%) immediately after a KC but independently of the features of this particular KC (Kokkinos and Kostopoulos, 2011). The sleep maintenance role is supported by the fact that damage to the dorsal and basal forebrain areas, which we show to be associated with spindles and KCs, are correlated with insomnia (Altena et al., 2010; Koenigs et al., 2010). Spindles and KCs provide controlled isolation along with synchronization of the brain for their duration (Colrain, 2005; Llinás and Steriade, 2006; Sato et al., 2007; Cash et al., 2009). During this time the brain can go to work uninterrupted and unbiased from environmental stimuli to accomplish its functions: restoration, economy, and especially memory consolidation.

In the case of spindles isolation from the environment has priority; it is facilitated in the pre-spindle period by modest increases in spectral power of low frequencies that inhibit any current frontal lobe action, including processing of external stimuli. During spindles prominent sigma band increases are established in the basal forebrain and in MPFC and posterior parietal areas suggesting facilitation of the dialogue between these areas, as would be expected by a process enabling the distribution of engrams from the temporary hippocampal store to the cortex (Siapas and Wilson, 1998; Clemens et al., 2011).

However, useful to sleep's roles, a complete sensory deprivation would make an animal vulnerable to predators and homeostatic emergencies. There is a need for a sentinel and KCs have been proposed to play such role at sleep's initial stages (the “Janus response,”; Jahnke et al., 2012; Halasz and Bodizs, 2013). KCs are proposed to be triggered by stimuli and contain a cognitive process evaluating the salience or threatening value of the stimulus and in the negative case a synchronizing and therefore hypnagogic influence to the brain. Our findings support this hypothesis: The regional increases in both low and high frequencies are seen only in the period before KCs (but not before spindles, Figure 5) and notably, in the rACC an area associated with detecting internal or external perturbations (Luu and Pederson, 2004). These activation patterns suggest that, in the case of KCs, some cognition (Gross and Gotman, 1999) and possibly alertness (to deal with potential danger) co-exist with active inhibition in the same brain areas.

The testable hypotheses we put forward above are only the first steps toward the description of an advanced refinement of the overall mechanism for gating perception during sleep employing both KCs and spindles. They also provide means of qualifying, testing and advancing modern theories about memory consolidation and the sentinel hypothesis (Jahnke et al., 2012; Halasz and Bodizs, 2013). Progress along this line of research could also provide powerful hints for understanding pathologies that relate to the high synchrony (found during spindles) and high amplitude (found during KCs) events that represent a shift closer to pathology (Si et al., 2010; Tezer et al., 2014).

### Limitations

The small number of subjects (four) is a possible **limitation** of this study and further studies with more subjects are needed to remove concerns about the generalizability of the results to other subjects. However, the methodology adopted here allows detailed tomographic and statistical analysis of individual subject data, producing very robust results, which are reproduced in each and every subject. More specifically, since the spectral frequency of spindles is stable for each subject but varies between subjects (Werth et al., 1997) we analyzed data separately for each subject and then applied stringent statistics to reveal commonalities in all four subjects.

A further limitation is that while KCs can also be elicited by sensory stimuli (Laurino et al., 2014), the present study examines only spontaneous KCs. Furthermore, our work indicates the main nodes of the circuits associated with NREM features, the times of their activation and the spectral content, but not the interactions between nodes and the overall properties of the network.

Recovery of deep sources from MEG and EEG has been the topic of hot disputes for decades and it may be a point of concern given that some of the generators we have identified are not superficial. Recent arguments on both sides of the divide are discussed in some detail in Section Source Reconstruction. Here we simply add that all the results reported here were obtained using MFT, a method that has been successfully used in many prior studies for estimating neural sources of MEG signals throughout the brain (Ioannides, 2006; Poghosyan and Ioannides, 2007, 2008; Ioannides and Poghosyan, 2012), including deep structures, such as the thalamus (Ribary et al., 1991), amygdala, cerebellum and brainstem (Ioannides et al., 2004a,b, 2005).

## Author contributions

AI conceived initiated and directed the study, for both the experimental phase at the RIKEN BSI and the analysis in Cyprus. AI, LL, and GK contributed to the initial experiment planning. AI and VP adapted analysis methods to specific needs of the study and together with LL performed the data analysis in Cyprus. All authors contributed to writing the paper.

### Conflict of interest statement

AI, LL, and VP worked for AAI Scientific Cultural Services Limited (AAISCS) for much of the work that lead to the publication and the first two continued to do so. The other author, GK, declares that the research was conducted in the absence of any commercial or financial relationships that could be construed as a potential conflict of interest.
